# TFEB, FOXO3 and TLR4 in resveratrol-induced autophagy in a mucopolysaccharidosis IIIB mouse model

**DOI:** 10.1038/s12276-026-01643-0

**Published:** 2026-02-05

**Authors:** Estera Rintz, Magdalena Podlacha, Lidia Gaffke, Grażyna Jerzemowska, Zuzanna Cyske, Karolina Pierzynowska, Grzegorz Węgrzyn

**Affiliations:** 1 Department of Molecular Biology, Faculty of Biology, University of Gdańsk, Gdańsk, Poland; 2 Department of Animal and Human Physiology, Faculty of Biology, University of Gdańsk, Gdańsk, Poland

**Keywords:** Molecular neuroscience, Macroautophagy, Mechanisms of disease

## Abstract

Mucopolysaccharidosis (MPS) type IIIB is the progressive degeneration of the central nervous system. Resveratrol is proposed as a potential therapeutic molecule as a drug reducing inflammation and for improving behavior of MPS mice. Here we investigated autophagy in correlation with immune response in an MPS IIIB mouse model. The effects of resveratrol on mouse behavior and the levels of selected cytokines that influence the inflammation were assessed. The study was performed on both male and female mice treated or not with resveratrol. The results of behavioral, molecular and biochemical experiments confirmed that autophagy and immune response are disturbed in MPS IIIB mice. A correlation between behavioral disturbances and levels of heparan sulfate and TLR4 could be observed. The FOXO3 transcription factor was identified as one of the key factors in the resveratrol-mediated stimulation of the autophagy process in the MPS IIIB mouse model, though it was not the sole pathway induced by this compound. We conclude that resveratrol can modulate the degradation of glycosaminoglycans and also may contribute to the reduction of inflammation and the normalization of animal behavior in the MPS IIIB model.

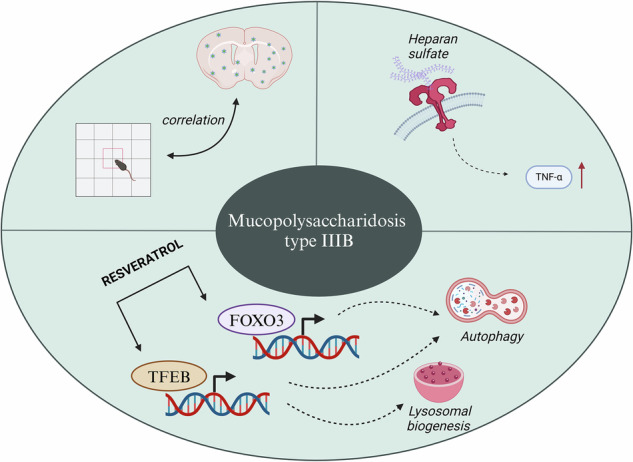

## Resveratrol enhances autophagy and behavior in MPS IIIB mice

Mucopolysaccharidosis (MPS) is a rare genetic disorder that affects the body’s ability to break down certain sugars. Sanfilippo disease, or MPS III, is particularly damaging to the brain. Here researchers explored the potential of resveratrol, a natural compound with anti-inflammatory properties, as a treatment. The study used a mouse model of MPS IIIB to test resveratrol’s effects. Mice were given resveratrol from 8 weeks old and monitored until 30 weeks. The researchers measured sugar levels in urine and conducted behavioral tests to assess improvements. They also examined how resveratrol affected inflammation and brain function. The results showed that resveratrol reduced sugar accumulation and improved behavior in both male and female mice. It also decreased inflammation in the brain and body. The study concluded that resveratrol could be a promising treatment for MPS III by enhancing autophagy and reducing inflammation.

This summary was initially drafted using artificial intelligence, then revised and fact-checked by the author.

## Introduction

Mucopolysaccharidosis (MPS) is a group of rare lysosomal storage diseases with an estimated overall incidence of approximately 1 in 100,000 births. Each MPS type is caused by a genetic defect resulting in the lack or severe deficiency of one of the enzymes involved in the degradation of glycosaminoglycans (GAGs). Based on the missing enzyme, 12 classical MPS diseases can be distinguished, two which can or cannot be classified as MPS, depending on the criteria used^1–3^. The progressive accumulation of GAGs results in multi-organ damage, causing severe symptoms^4,5^. The clinical manifestations of MPS include neurological symptoms, developmental delay, cognitive impairment, hyperactivity and other behavioral changes, sleep disorders, facial dysmorphia, vascular diseases, skeletal disorders, respiratory problems and visual disturbances; the average life span of a person with MPS is between one and two decades^6,7^.

Sanfilippo disease, also classified as MPS III, is characterized by especially severe neuronopathic defects. Among the subtypes of this disease determined in humans, subtypes IIIA and IIIB occur more frequently than IIIC and IIID^8^. The course of Sanfilippo disease can be divided into three phases. Phase one begins between the ages of 1 and 3 years and manifests in development and speech delay^9^. Phase two occurs between the ages of 3 and 4 years. The symptoms are progressive developmental impairment, sleep disturbances and behavioral changes, such as hyperactivity and anxiety. The third phase begins at adolescence, and problems include swallowing and memory impairment. Owing to progressive GAG accumulation, the disease progress leads to stiffness of muscle and a decline in motor skills, with the patient ultimately being in a vegetative state. In the last stage of the disease, patients are extremely affected and rarely live more than two decades^10–13^.

Currently, there is no effective therapy available for patients with MPS III^7,14^. It seems that delivery of the missing enzyme, using enzyme replacement therapy, would be the best treatment option for patients. However, enzyme replacement therapy has some limitations. First, the enzyme molecule cannot cross the blood–brain barrier, thus it is not able to correct central nervous system (CNS) pathology^15^. Second, it is an expensive therapy (up to US$600,000 per year for a 25 kg patient^16^). Third, many patients have an immune response against the enzyme^15^. Another potential therapy is transplantation of hematopoietic stem cells that, owing to cells’ differentiation potential to microglia, could correct CNS pathology. Unfortunately, because of graft-versus-host disease and difficulty in finding a suitable donor, the therapy is connected to many complications with low to no effectivity in patients with MPS III^16–19^. Thus, neither of these therapies discussed above are able to correct neurological symptoms in Sanfilippo disease.

Most recent studies have focused on the correction of the immune response that potentially may influence neurological outcomes of the disease^20^. Increased GAG accumulation in the lysosome affects its proper function, which leads to autophagy impairment, mitochondrial disturbance, oxidative stress and ultimately high inflammation in the periphery and in the CNS. Heparan sulfate (HS; a GAG that is accumulated in MPS III) binds to Toll like receptor 4 (TLR4)^21,22^, which overactivates the innate immune response. This response is correlated with an increased risk of graft-versus-host disease in patients with MPS III^21^.

In light of this, resveratrol, a small molecule with many biological properties, could potentially reduce the inflammation in both peripheral and CNS^23^. Resveratrol (3,5,4′-trihydroxy-*trans*-stilbene or 5-[(*E*)-2-(4-hydroxyphenyl)ethen-1-yl]benzene-1,3-diol) belongs to the group of polyphenolic derivatives of stilbenes, naturally occurring substances that have many biological functions^24^. The biological activities of resveratrol include antioxidative, anti-inflammatory, anticancer, anticoagulant, antithrombotic and antiviral actions, which have been comprehensively reviewed recently^25^. Moreover, the neuroprotective effects, including the prevention of cognitive decline, of this compound have been reported^26–30^. These properties, in combination with documented stimulation of the autophagy process, which appears to be beneficial in MPS (as reviewed recently^31^), led to the hypothesis that resveratrol might be a potential drug for MPS.

Since Sanfilippo disease is characterized by especially severe neuronopathy and no treatment of this condition is currently available, we aimed to test the hypothesis on the potential therapeutic effects of resveratrol. Notably, our recent comparative study identified resveratrol as the most effective agent among several natural compounds—including genistein, curcumin, trehalose and capsaicin—in reducing HS accumulation in fibroblasts derived from patients with MPS III^32^. With use of the male MPS IIIB mouse model, we demonstrated that this compound can effectively reduce GAG levels and correct the behavior of affected animals, mainly due to stimulation of the autophagy process^32^. These findings highlighted resveratrol’s dual therapeutic potential to reduce pathological HS storage and ameliorate neurological dysfunction, making it a potential candidate for Sanfilippo syndrome (MPS III) treatment. However, the specific mechanism of resveratrol-mediated autophagy induction in MPS III remained unclear, as were the effects on the immune response and any sex-related differences. In particular, the specific pathways through which resveratrol induces autophagy, its interaction with immune responses—especially TLR4-mediated signaling—and the influence of sex on treatment outcomes are not yet fully understood. Moreover, most previous studies have been limited to male models, leaving the response in females largely unexamined. Therefore, in this work, we used a mouse model of MPS IIIB with both sexes, bearing knockout mutations in both alleles of the *Naglu* gene (disruption of the part of exon 6), encoding α-*N*-acetylglucosaminidase (NAGLU)^33^. This study was designed to clarify the molecular pathways by which resveratrol activates autophagy in MPS IIIB, distinguish whether these effects are mediated through mTOR-dependent or independent mechanisms and explore how these processes are linked to inflammation. In parallel, we investigated whether the therapeutic response to resveratrol differs between sexes and how these differences correlate with changes in immune activation and pathological progression. Molecular studies were performed using both mouse and cellular (mouse-derived fibroblasts) models. This study demonstrates, in a comprehensive manner, the dysregulation of autophagy in MPS IIIB mice and the mechanism of its correction by resveratrol, with emphasis on the correlation of the regulatory processes to the immune response and sex of animals.

## Materials and methods

### Mouse model

All experiments were conducted with male and female homozygous *Naglu*-knockout mice (MPS IIIB) with the targeted mutation in the gene coding for NAGLU (B6.129S6-Naglu^tm1Efn^/J; 003827; The Jackson Laboratories) and their healthy littermates (wild type (WT); C57BL/6) as controls. The mice were kept in a ventilated animal facility, constant artificial lighting cycle (12 h light/12 h dark), ambient temperature (22 °C) and constant air humidity (55%). Access to food and clean water was ad libitum.

All experiments were carried out with consent of the Local Ethics Committee for Animal Experiments in Bydgoszcz, Poland (no. 13/2020 on 25 June 2020). The animal house meets the requirements of the Act on Animal Experiments of 21 January 2005 (Journal of Laws of 24 February 2005), ordinances of the Minister of Agriculture and Development and the provisions of March 10, 2006 (Journal of Laws of 28 March 2006), as well as the recommendations of the European Commission on the welfare of animals used in scientific experiments (Tricity Academic Experimental Animal House).

### Experimental groups and the resveratrol dose

**Fig. 1:**
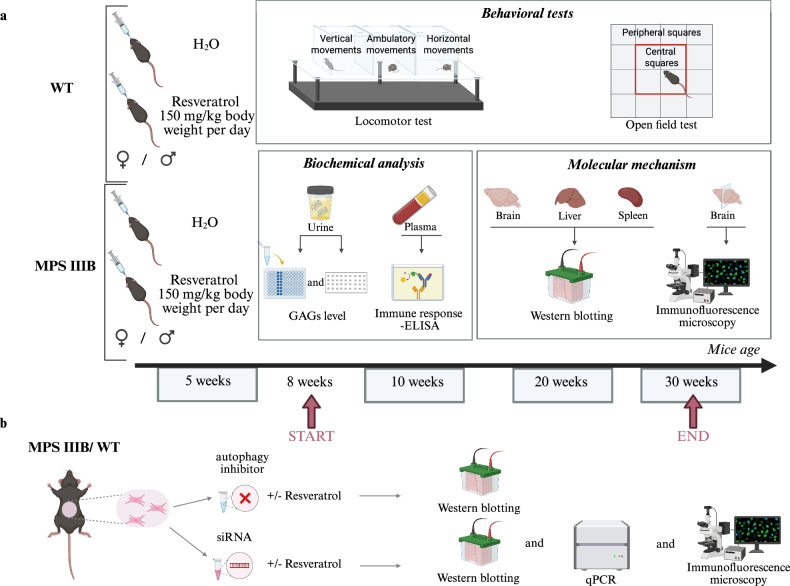
The experimental design. **a** Experiments started when mice were 5 weeks old. Resveratrol or water was administered daily though oral gauge from 8 weeks. During the experiments, at four time points, behavioral and biochemical analyses were performed. Animals were killed after the last point at the age of 30 weeks, and molecular analyses of samples were performed. **b** WT or MPS IIIB fibroblast cells were isolated from mice to perform inhibition of autophagy and silencing of genes. Images in panels **a** and **b** created in BioRender.com.

Mice were separated in eight experimental groups (four male groups and four female groups), described as (1) WT mice treated with water (WT group), (2) WT mice treated with resveratrol at a of dosage 150 mg/kg body weight per day (WT + R group), (3) MPS IIIB mice treated with water (MPS IIIB group) and (4) MPS IIIB mice treated with resveratrol at a dosage of 150 mg/kg body weight per day (MPS IIIB + R group). Figure 1a shows the experimental design for the mouse experiment. Resveratrol (Sigma-Aldrich) suspensions in water were administered orally though metal gavages at a dosage of 150 mg/kg body weight per day from 8 weeks of age (the average weight of animals at this stage was 15 ± 2 g). This dosage was selected based on our previous study, in which 50 mg/kg/day resveratrol effectively improved behavioral and biochemical outcomes in male MPS IIIB mice^32^, but failed to show therapeutic efficacy in females under the same conditions (Supplementary Fig. 1). Therefore, to achieve comparable therapeutic effects across both sexes in the current study, the dosage was increased to 150 mg/kg/day. Although resveratrol is known to cross the blood–brain barrier^34^, its brain penetration is limited due to rapid metabolism and systemic clearance, primarily via glucuronidation and sulfation^35,36^. Pharmacokinetic studies in rodents have reported that oral bioavailability of resveratrol is low (<20%), with a short plasma half-life (~14 min)^37^. It was reported that doses ranging from 100 to 250 mg/kg/day should be used in preclinical models to ensure sufficient CNS exposure^32,38–45^. While lower doses (for example, 500–1,000 mg/day) of resveratrol were employed in human clinical trials^41,46^, these are unlikely to reach therapeutic brain concentrations owing to the pharmacokinetic limitations. Consequently, higher dosing in animal studies remains a justified approach for investigating CNS-targeted therapeutic effects of resveratrol.

Genotyping was performed on 21-day-old mice for the *Naglu* gene by PCR with the forward primer 5′-GTC GTC TCC TGG TTC TGG AC-3′ and one of two reverse primers, for either the WT allele 5′-ACC ACT TCA TTC TGG CCA AT-3′ or the mutant allele 5′-TGG ATG TGG AAT GTG TGC GAG-3′. A master mix of phusion polymerase (Thermo Fisher Scientific) was used with the following reaction conditions: 94 °C, 3 min; 30 cycles of 94 °C for 40 s, 65 °C for 30 s and 72 °C for 45 s; 72 °C for 5 min.

### Locomotor activity measurement in actometers

Locomotor activity was measured at four time points: 5, 10, 20 and 30 weeks of age using actometers (Opto Varimex Minor) with dimensions 43 × 43 × 20 cm. The movement of the animals was detected by the photocell, which was recorded by an automatic counter. Three movements were analyzed: vertical, horizontal and ambulatory (for example, grooming). Animals were not trained for the behavioral test before performance. The measurements were made between 15:00 and 16:00 and lasted 10 min.

### The open field test

The open field test assessment was conducted at four different time points, similar to locomotor activity evaluations. The open field test took place in a white, wooden arena enclosed by low walls, measuring 100 × 100 × 60 cm, with the floor divided into 25 squares (central and peripheral). Each session lasted 15 min and was recorded using EthoVision XT 10 software (Noldus). Mice were introduced to the arena facing a corner. To prevent scent-based interference, the surface was cleaned with a 70% ethanol solution and allowed to dry between trials. Key behavioral parameters analyzed included time spent in central versus peripheral squares, exploration time (rearing behavior, where mice stand on their hind legs to explore) and freezing time (duration of immobility). The animals did not undergo prior training for the behavioral test. Measurements were consistently conducted between 15:00 and 16:00.

### Glycosaminoglycan analysis

The urine of mice was collected at four time points: after 5, 10, 20 and 30 weeks of the supplementation. Urine collection was made with the use of the hydrophobic sand (LabJot) to reduce the stress of the animals. Total GAG level was measured using the Blyscan Glycosaminoglycan Assay commercial kit, where 1,9-denethylmethylene blue (DMMB) binds to the GAG sulfone groups. The effects of this colorimetric reaction were then measured using a spectrophotometer.

In addition, HS was measured with dot-blot in the urine of 30-week-old mice. The procedure was as follows: after lysis and the determination of protein concentration with the BCA protein assay kit (23225, Thermo Fisher Scientific), the protein extracts were fixed directly to the membrane using a dot/slot-blot apparatus (Bio-Rad). The membrane was blocked with 5% non-fat milk in PBS-T buffer (phosphate-buffered saline with Tween 20 BioShop; 10010023, Thermo Fisher Scientific). After that, monoclonal antibodies against HS (NBP2-23523, Novus Biologicals) were added at a dilution of 1:500 and incubated overnight in 4 °C. Following the wash, the membrane was incubated with secondary antibody (A5420 mouse, Sigma-Aldrich) at room temperature (20–25 °C) for 1 h. Then the substrate (Clarity Western ECL Substrate, 500 ml 1705061, Bio-Rad) was added and membrane was exposed to an X-ray film to detect the signal. Analysis was performed with ImageJ 1.54i software.

### Analysis of selected cytokines

To quantify the concentrations of tumor necrosis factor (TNF) and interleukin-10 (IL-10) in plasma, an enzyme-linked immunosorbent assay (ELISA) was performed following the manufacturer’s protocol (Sigma-Aldrich). In summary, plasma samples obtained through centrifugation were thawed and 100 µl of each sample was added in duplicate to antibody-coated plates (specific for either TNF or IL-10). The plates were covered and incubated at room temperature for 1 h (IL-10) or 2 h (TNF). After incubation, the plates were washed three times before adding 100 µl of secondary antibodies. A second incubation followed, lasting 1 h, after which the plates were washed three times (IL-10) or five times (TNF). Subsequently, 100 µl of the substrate solution was added and the reaction proceeded for 30 min before being stopped. Absorbance was measured within 5 min of stopping the reaction using an EnSpire microplate reader (PerkinElmer) with EnSpire Manager software.

### Brain immunofluorescence

Immunofluorescence studies were performed on sections of the right half of the mouse brains, sliced into 20-μm-thick preparations using a Leica CM 1950 cryostat (Leica). The sections were placed on gelatin-coated microscope slides (Superfrost Plus Adhesion Microscope Slides with Tab; Epredia). Immunofluorescence staining was conducted to visualize the expression of c-fos (density of c-fos-positive cells), DAPI, TNF and IL-10.

All steps of the double-staining procedure were carried out at room temperature using PBS (pH 7.4), normal goat serum (NGS; Sigma-Aldrich; 5% NGS containing 0.3% Triton X-100) and 0.5% bovine serum albumin (BSA; Sigma-Aldrich). The first stage of immunofluorescence staining involved a 48-h incubation with a solution containing primary antibodies: mouse monoclonal IgG c-Fos antibody (c-Fos Antibody, dilution 1:200; Santa Cruz Biotechnology), rabbit monoclonal TNF antibody (anti-TNF antibody, dilution 1:100; Abcam) and rabbit polyclonal IL-10 antibody (anti-IL-10 antibody, dilution 1:100; Abcam), along with PBS, 0.3% Triton X-100 and 3% NGS. The next step involved a 4-h incubation with a mixture of secondary antibodies at a 1:500 dilution (CF543 goat anti-mouse IgG, spectrally similar to Alexa Fluor 546; Invitrogen, Thermo Fisher Scientific; CF488A Goat Anti-Rabbit IgG, spectrally similar to Alexa Fluor 488; Invitrogen, Thermo Fisher Scientific). The staining process concluded with mounting the samples using a hard-setting resin combined with DAPI (EverBrite Hardset Mounting Medium with DAPI; Biotium) and covering them with coverslips (24 × 60 mm, Bionovo).

Fluorescent images were acquired using a PrimoStar microscope (three-channel system) (Carl Zeiss MicroScopy GmbH) at magnifications of 4× 10 and 20× 10. All images were labeled with a white scale bar on a black background, in grayscale ranging from 0 (black) to 255 (white), and processed using Carl Zeiss Imaging Systems software (Axio Vision Rel. 4.9.1, Zeiss).

The boundaries of brain structures were determined based on the mouse brain atlas using contour traces from templates. The software calculated the specified area of brain structures (thresholding procedure), where optical density and size filters were calibrated to count white grains (>70% white) exceeding 15 pixels in size (15 μm²). This ensured that the program excluded all particles inconsistent with the size of the cell nucleus (c-Fos/DAPI) or cells containing TNF/IL-10 under the given magnification. Subsequently, the density of c-Fos-positive cells (c-Fos^+^), DAPI-positive cells (DAPI^+^) and TNF-positive (TNF^+^) or IL-10-positive (IL-10^+^) cells was counted as the number of nuclei or cells per mm² of the analyzed structure.

Microscopic analysis was conducted in sections on a fixed rostral–caudal level to Bregma for each structure in select limbic brain regions. The analysis including the limbic cortex, specifically the cingulate cortex, area 1 (CG1) and cingulate cortex, area 2 (CG2): Bregma from +0.98 mm to +0.26 mm; retrosplenial cortex, agranular part (RSA) and granular part (RSG): Bregma from −0.94 mm to −1.22 mm, as well as in the amygdala (Amg) region of the brain: central nucleus of the amygdala (Ce) and basolateral nucleus of the amygdala (BLA): Bregma from −0.94 mm to −1.06 mm.

### Reagents

Resveratrol (*trans*-3,4′,5-trihydroxystilbene; 1,3-benzenediol, 5-[2-(4-hydroxyphenyl)ethenyl]) (sc-200808B, Santa Cruz Biotechnology) was prepared every day in water as a solution and given to mice at dosage of 150 mg/kg body weight. Cells were treated with resveratrol at dose 80 µM, stock solutions were prepared in dimethyl sulfoxide (DMSO) and stored at −20 °C. Bafilomycin A1 (J61835, Thermo Fisher Scientific) was dissolved in DMSO and cells were treated with this compound at final concentration of 100 nM.

The primary antibodies used for this study were as follows: anti-LC3B sc-376404, anti-p62/SQSTM1 sc-28359 (Santa Cruz Biotechnology); anti-TFEB 37785, anti-P-TFEB 87932S, anti-4EBS6K 9202S, anti-P-4EBS6K 9234S, anti-SIRT1 9475S, anti-P-SIRT1 2314S, anti-AMPKα 2532S, anti-TLR4 14358S, anti-EIF4EBP1 9452S, anti-P-EIF4EBP1, anti-Beclin-1 3495S (Cell signaling Technology); anti-ACTB MA1-744, anti-IRAK 8F1A7, anti-P-IRAK bs-3193R, anti-P-TLR4 PA5-105713 (Thermo Fisher Scientific); and anti-FOXO3 SP0054, anti-P- FOXO3 ST4901 (Novus).

The secondary antibodies used for this study were as follows: Alexa Fluor 555, A-21428, A-21422 (Invitrogen, Thermo Fisher Scientific); and western blotting: A0545 rabbit or A5420 mouse (Sigma-Aldrich).

### Cell lines and cell cultures

Primary fibroblasts were isolated from both MPS IIIB and WT mice. Briefly, each mouse was euthanized and all procedures for skin isolation were carried out under sterile conditions within a laminar flow hood. The mice were disinfected with 70% ethanol and their fur was removed using a razor blade. After another disinfection event, abdominal skin was excised using sterilized scissors. The collected tissue was rinsed with PBS containing antibiotics. Portions of the tissue were then minced and placed in 6-well plates containing Dulbecco’s modified Eagle medium (DMEM) (Thermo Fisher Scientific), supplemented with 10% fetal bovine serum (FBS) (Thermo Fisher Scientific) and 1% antibiotic/antimycotic solution (Sigma-Aldrich). The plates were incubated at 37 °C in the chamber with 5% CO_2_. After 3 days, primary fibroblasts had migrated onto the plate and the remaining tissue was removed. The cells were tested for mycoplasma contamination using the Mycoplasma PCR Kit (E0448, EURx), following the manufacturer’s instructions. All experiments were conducted using cells at passages 4–8.

### Autophagy inhibition

Autophagy was inhibited using Bafilomycin A1 (BafA1). Cells (a total number of 5 × 10⁵) were seeded onto 10 cm culture dishes in complete DMEM supplemented with FBS and antibiotics. After 24 h, BafA1 was added to the medium at a final concentration of 100 nM. Two hours later, resveratrol (80 µM) was added to the culture. After 24 h of incubation, cells were collected for western blot analysis of the LC3 protein level.

### Gene silencing

The siRNA-mediated silencing of gene expression in mouse fibroblasts was performed according to a previously described procedure^47^ with minor modifications. Lipofectamine RNAiMAX Transfection Reagent (13778075, Thermo Fisher Scientific) was used for transfection, according to the manufacturer’s instructions. siRNAs were specific for either mouse FOXO3 (siRNA ID: 433579, 433577) or TLR4 (siRNA ID: 188775, 188776) (4390771, Thermo Fisher Scientific).

To measure mRNA levels of the selected genes (*FOXO3* and *TLR4*), RT–qPCR was performed. Briefly, 1.7 × 10^5^ cells were plated onto 6-well plate and reverse transfection was performed according to the manufacturer’s instructions. After 24 h, resveratrol (80 µM) was added to the cell culture and cells were incubated overnight at 37 °C with 5% CO_2_. RNA was isolated using commercially available kit (RNeasy Micro Kit 74004, Qiagen 217084). cDNA was transcribed with the EURx kit (E0801, EURx). To perform gene quantification, we used the TaqMan Gene Expression Assay (20×) and used the TaqMan Fast Advanced Master Mix for PCR reaction (4444557, Thermo Fisher Scientific). The results are shown as the relative expression to the control *GAPDH* gene.

### Immunofluorescence

Microscopic immunofluorescence analysis of target proteins was carried out based on established protocols^48^. In summary, 4 × 10⁴ cells per well were seeded onto coverslips in a 12-well plate and allowed to adhere overnight. After a 48-h treatment with resveratrol and/or siRNA, the cells were fixed with 2% paraformaldehyde in PBS and underwent subsequent washing steps. To prevent nonspecific binding, blocking was performed using BSA (A7906, Sigma-Aldrich). The cells were then incubated with primary antibodies overnight. Following further washes, secondary antibodies were applied for 1 h. After additional washing, coverslips with cells were mounted onto slides using ProLong Gold Antifade Mountant with DAPI (P36935, Invitrogen, Thermo Fisher Scientific). Fluorescence microscopy was performed using an Olympus microscope and data analysis included mean fluorescence intensity and colocalization, assessed with CellSens software. Statistical evaluation involved capturing 12–30 images per treatment from four distinct cell lines, encompassing both MPS IIIB and healthy control (WT) samples. Images, captured at 100× magnification, feature a 10-μm scale bar.

### Immunoblotting

After lysis and determination of protein concentration with the BCA protein assay kit (23225, Thermo Fisher Scientific). The levels of proteins of interest were determined by either a traditional or an automatic western blotting, based on previously published experimental procedures^48^.

In most experiments, proteins were separated employing the WES system (automated western blots with simple western; ProteinSimple) as previously described^47^, with a 12–230 kDa separation module (SM-W003) and detected with the anti-mouse detection module (DM-002) or anti-rabbit detection module (DM-001, ProteinSimple), according to the manufacturer’s instruction. The total protein level, determined using a total protein detection module for chemiluminescence (DM-TP01, ProteinSimple), was used as the loading control. As not all proteins can be effectively shown with the WES system, for TFEB and LC3-II we used regular western blotting.

In the regular western blotting, protein extracts were separated by SDS–PAGE electrophoresis. Following the electrophoresis, the proteins were transferred to the membrane with the Iblot3 system (Thermo Fisher Scientific). The membrane was blocked with 5% non-fat milk in the PBS-T buffer (Tween 20, BioShop; PBS 10010023, Thermo Fisher Scientific) and then incubated with primary antibody overnight at 4 °C. After reactions with primary antibodies, the membrane was washed with PBS-T and incubated with secondary antibody (A0545 rabbit or A5420 mouse, Sigma-Aldrich) at room temperature for 1 h. Then, the substrate (Clarity Western ECL Substrate, 500 ml 1705061, Bio-Rad) was added and the membrane was exposed to the X-ray film to detect the signal. Western blotting analysis was performed with ImageJ 1.54i software.

In most experiments, proteins were separated employing the WES system (ProteinSimple), with a 12–230 kDa separation module (SM-W003) and detected with anti-mouse detection module (DM-002) or anti-rabbit detection module (DM-001, ProteinSimple), according to the manufacturer’s instruction. The total protein level, determined using a total protein detection module for chemiluminescence (DM-TP01, ProteinSimple), was used as the loading control.

### Statistical analysis

The Kolmogorov–Smirnov test was used to evaluate the normality of data distribution, while the Levene test assessed the homogeneity of variance. Depending on these results, analysis of variance (ANOVA) followed by Tukey’s post hoc test was performed for normally distributed data. If the assumptions of normality and homogeneity of variance were not met, the nonparametric Kruskal–Wallis test was applied, followed by Dunnett’s test for multiple comparisons. All statistical analyses were conducted using GraphPad Prism 9 software, with statistical significance set at *P* < 0.05. Error bars in the figures represent the standard deviation, as specified in the figure legends. Statistically significant differences are indicated by asterisks: *P < 0.05, **P < 0.01, ***P < 0.001 and ****P < 0.0001. The results obtained for in MPS IIIB males and females at final weeks were analyzed with a *t*-test and statistically significant differences (*P* < 0.05) are indicated with hashtags (#).

## Results

### Mouse model of MPS IIIB and experimental design

As an animal model of MPS IIIB, we used *Naglu*-knockout mice with the homozygous mutation in the gene coding for NAGLU and WT control mice. Males and females were investigated as separate groups to test for any sex-related differences. For in vitro experiments, skin fibroblasts were obtained from MPS IIIB and control mice, and cultures of these cell lines were established. A scheme of the experiments is shown in Fig. 1. Either resveratrol (at a dosage of 150 mg/kg/day) or water was administered orally to animals starting at the age of 8 weeks, and the experiments were terminated when animals reached 30 weeks. The in vivo experiments involved urinary GAG level measurements, behavioral tests, determination of immune response markers in blood and molecular studies on the mechanism of resveratrol action, including estimation of molecular markers of autophagy and other processes in various organs. The in vitro experiments included experiments with cultures of mouse fibroblasts, especially determining the levels of various molecular markers, after using different manipulations such as the addition of specific inhibitors or using siRNAs to silence the expression of selected genes.

### Resveratrol supplementation reduces urinary GAGs

**Fig. 2:**
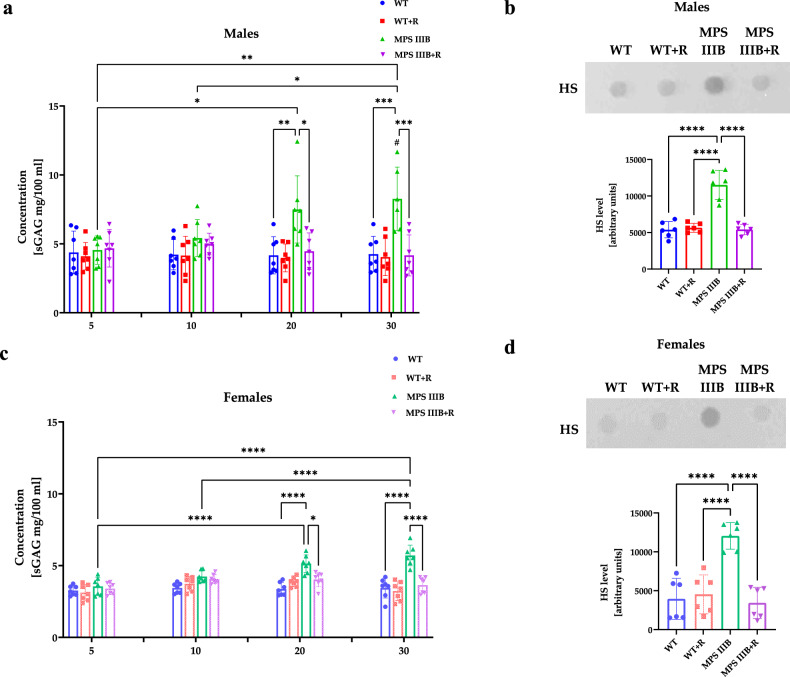
Accumulation of urinary GAGs in control (WT) and MPS IIIB mice. **a** The amount of urinary total sulfated GAG (sGAG) in male mice. **b** The amount of HS in male mice. **c** The amount of urinary sGAG) in female mice. **d** The amount of HS in female mice. The results are shown as mean values ± s.d. (*n* = 6–7). Statistically significant differences are indicated by asterisks: *P < 0.05, **P < 0.01, ***P < 0.001 and ****P < 0.0001, #males versus females MPS IIIB *P* < 0.05.

**Fig. 3:**
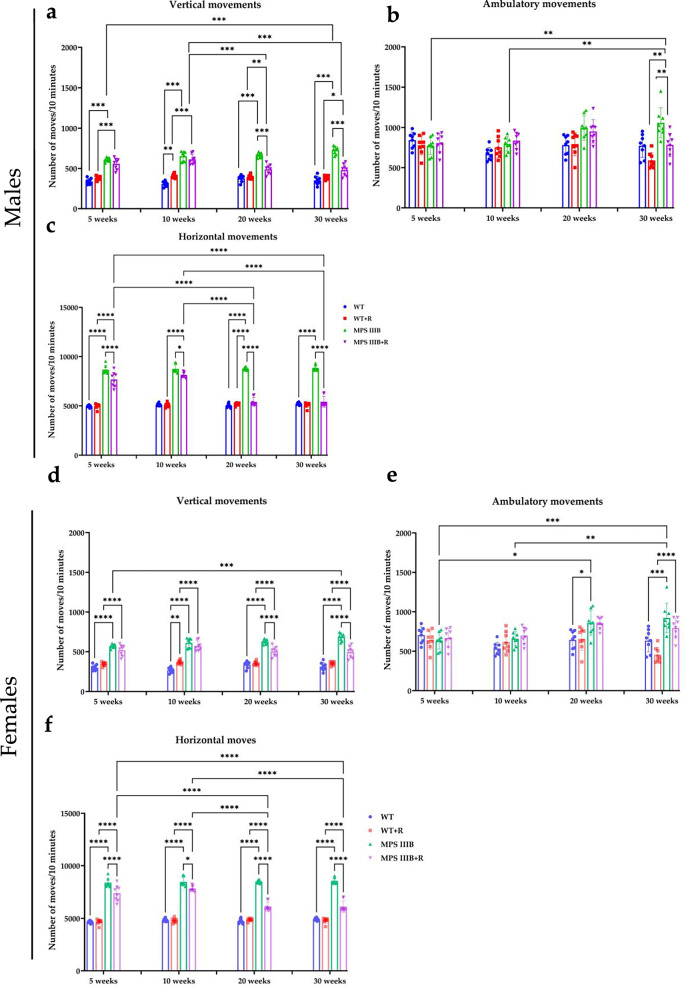
Locomotor analysis of control (WT) and MPS IIIB mice. **a** The number of vertical movements in male mice. **b** The number of ambulatory movements in male mice. **c** The number of horizontal movements in male mice. **d** The number of vertical movements in female mice. **e** The number of ambulatory movements in female mice. **f** The number of horizontal movements in female mice. The results are shown as mean values ± s.d. (*n* = 6–7). Statistically significant differences are indicated by asterisks: *P < 0.05, **P < 0.01, ***P < 0.001 and ****P < 0.0001.

Accumulation of GAGs is the principal feature of MPS, and one of GAGs, HS is stored in MPS III^1^. Therefore, we measured urinary GAG levels as the primary biochemical MPS IIIB marker in mice. Elevated urinary HS levels were evident in both males and females of MPS IIIB mice, but only from 20 weeks of age (Fig. 2), whereas some of the symptoms detected in the behavioral analysis started before 20 weeks of age (Fig. 3). GAG accumulation starts in MPS cells from the very beginning of life, while elevated urinary GAG levels arise as a result of the exfoliation of the epithelium in the urinary tract and the release of GAGs from damaged cells^49^. Therefore, detectable elevation of urinary GAGs requires more time than cellular storage of these compounds, which can affect cellular functions. Importantly, administration of resveratrol at 150 mg/kg/day, starting at week 8, resulted in a decrease of urinary HS levels in MPS IIIB males and females to the level of WT mice (Fig. 2). In male mice, the total urinary GAG level at the final week was reduced from 8.26 ± 2.3 mg/100 ml in the MPS IIIB group to 4.16 ± 1.48 mg/100 ml in the MPS IIIB + R group (Fig. 2a). In female mice, the corresponding level decreased from 5.71 ± 0.71 mg/100 ml to 3.66 ± 0.55 mg/100 ml (Fig. 2c). These results indicated that total GAG accumulation at the final time point was higher in males when compared individually (*P* = 0.0246, *t* = 2.644); however, resveratrol effectively reduced GAG levels in both sexes. These results corroborated the conclusion about the efficacy of resveratrol in correction of the primary MPS IIIB biochemical defect.

### Resveratrol supplementation normalizes hyperactivity resulting from high levels of anxiety in male and female MPS IIIB mice

Patients with MPS IIIB are characterized by severe behavioral disturbances and progressive loss of cognitive and motor functions^50,51^. The phenotype of MPS IIIB mice closely resembles that of human patients and can be assessed using behavioral tests^52^. The effect of resveratrol over time was evaluated across all mouse experimental groups using two behavioral assessments: the locomotor activity test and the open field test. When testing locomotor activity, three types of movements were analyzed: vertical, ambulatory and horizontal. Interestingly, abnormalities in the physical activity were already evident in MPS IIIB mice (both males and females) at the age of 5 weeks (the age at which elevated urinary GAG excretion is not yet observed), especially for vertical (Fig. 3a, d) and horizontal movements (Fig. 3c, f), while changes in ambulatory movement (Fig. 3b, e), relative to control mice, were observed at later phases of the experiment. Hyperactivity, measured by horizontal movements, was comparable between male (8,555 ± 266 movements/10 min) and female (8,552 ± 266 movements/10 min) MPS IIIB mice during the final week, indicating no significant sex differences in this parameter. The applied behavioral analysis at appropriately selected time points allowed for the noninvasive monitoring of behavioral pattern abnormalities that, in the mouse model used, occur before the accumulation of glycosaminoglycans in urine, the levels of which are not significantly increased until around week 20. However, significant neuropathological changes occurring within CNS, which include GM2 and GM3 ganglioside accumulation, vacuolization of the lysosomes, microglia and astrocytes activation, as well as intense inflammatory process, which are already detected in the brain around 6 weeks of age in mice and increase with disease progression^53^.

Treatment with resveratrol (initiated at the age of 8 weeks) at the dosage of 150 mg/kg/day either improved (at earlier times) or corrected (at later times) the frequency of all types of movements in both male and female MPS IIIB mice (Fig. 3). These results confirmed that resveratrol is effective in improving/correcting behavior of MPS IIIB mice in males (as demonstrated previously with the use of a lower dosage of 50 mg/kg/day)^32^ and indicated that this compound can be equally effective in females, which was not the case with the lower dosage (Supplementary Fig. 1). It is noteworthy that the improvement included not only the correction of hyperactivity that, as indicated by the results obtained in the open field test, is due to high sensitivity to stress factors, but also included a reduction in the number of ambulatory movements that relate to different types of stereotypies observed not only in animals of both sexes, such as head shaking, licking or repeated body cleaning, but also in human patients^51^.

**Fig. 4:**
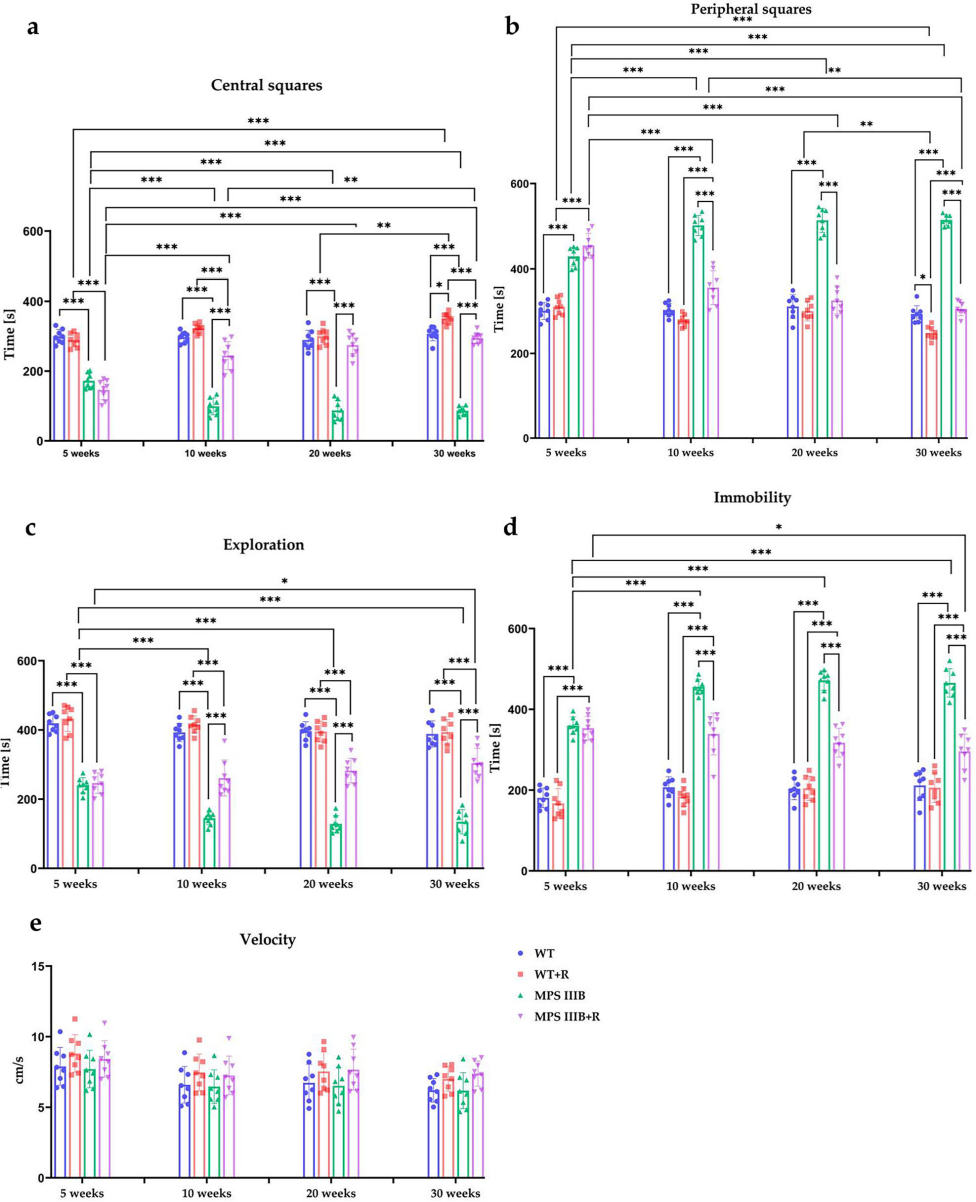
Locomotor activity and anxiety-related behaviors in the open field test of female control (WT) and female MPS IIIB mice. **a**–**d** Analysis of the time spent in central squares (**a**) the time spent in peripheral squares (**b**) the exploration time (**c**) the immobility time (**d**) and the velocity (**e**). The results are shown as mean values ± s.d. (*n* = 8). Statistically significant differences are indicated by asterisks: *P < 0.05, **P < 0.01, ***P < 0.001 and ****P < 0.0001.

**Fig. 5:**
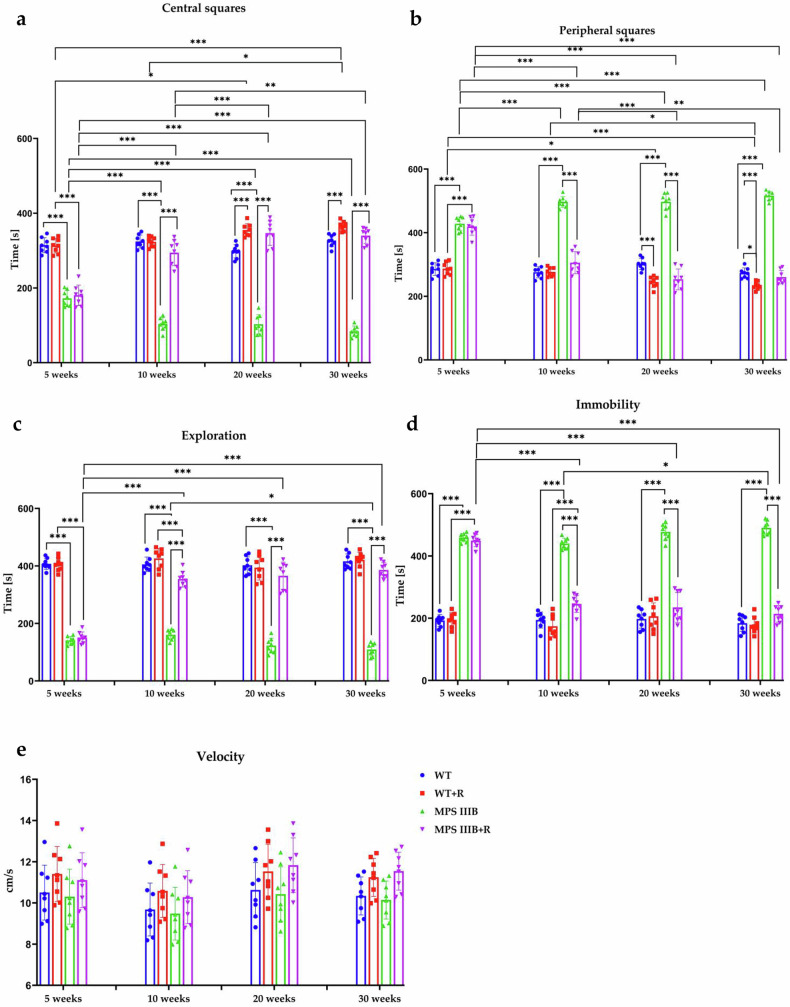
Locomotor activity and anxiety-related behaviors in the open field test of male control (WT) and male MPS IIIB mice. **a**–**d** Analysis of the time spent in central squares (**a**) the time spent in peripheral squares (**b**) the exploration time (**c**) the immobility time (**d**) and the velocity (**e**). The results are shown as mean values ± s.d. (*n* = 8). Statistically significant differences are indicated by asterisks: *P < 0.05, **P < 0.01, ***P < 0.001 and ****P < 0.0001.

The analysis of the animals’ behavior in the open field test showed that in male and female MPS IIIB mice, sensitivity to stress factors (bright light and open space) increased significantly as the disease progresses, as manifested by the shortened time the animals spend in the central squares and the prolonged time they spend in the safe zone, that is, peripheral squares (Figs. 4 and 5). A strong stress response disrupts the natural desire to explore the environment, which translates into a higher percentage of time spent in immobility. This disturbance was particularly pronounced in females that were spending statistically more time in immobility (Fig. 4d) each week, with the final week mean time of 465 ± 35 s compared with WT females that were immobile for 211 ± 37 s. In the case of male mice immobility (Fig. 5d), there was no difference between weeks (male MPS IIIB mice spent similar time in immobility in weeks 5 and 30 of the experiment), but there were significant differences between MPS IIIB and WT animals, for example, in the final week, immobility lasted 490 ± 22 s in MPS IIIB and 184 ± 26 s in WT mice. Resveratrol therapy eliminated these abnormalities in animals of both sexes. The therapeutic potential was particularly evident in males, which at 30 weeks of age practically did not differ from the control group in terms of the analyzed parameters (Fig. 5). In females, significant differences remained between the WT + R and MPS IIIB + R groups across all parameters. However, notable improvement was observed as all parameters were significantly improved in the MPS IIIB + R group compared to the untreated MPS IIIB group (Fig. 4). The lack of significant differences in the velocity allowed us to conclude that the observed differences between resveratrol-supplemented and untreated animals were not due to a lack of motivation or motor problems preventing movement, but confirmed the effectiveness of the tested compound.

### Resveratrol reduces levels of peripheral and central inflammatory markers without disturbing cytokine balance and neuronal activity

**Fig. 6:**
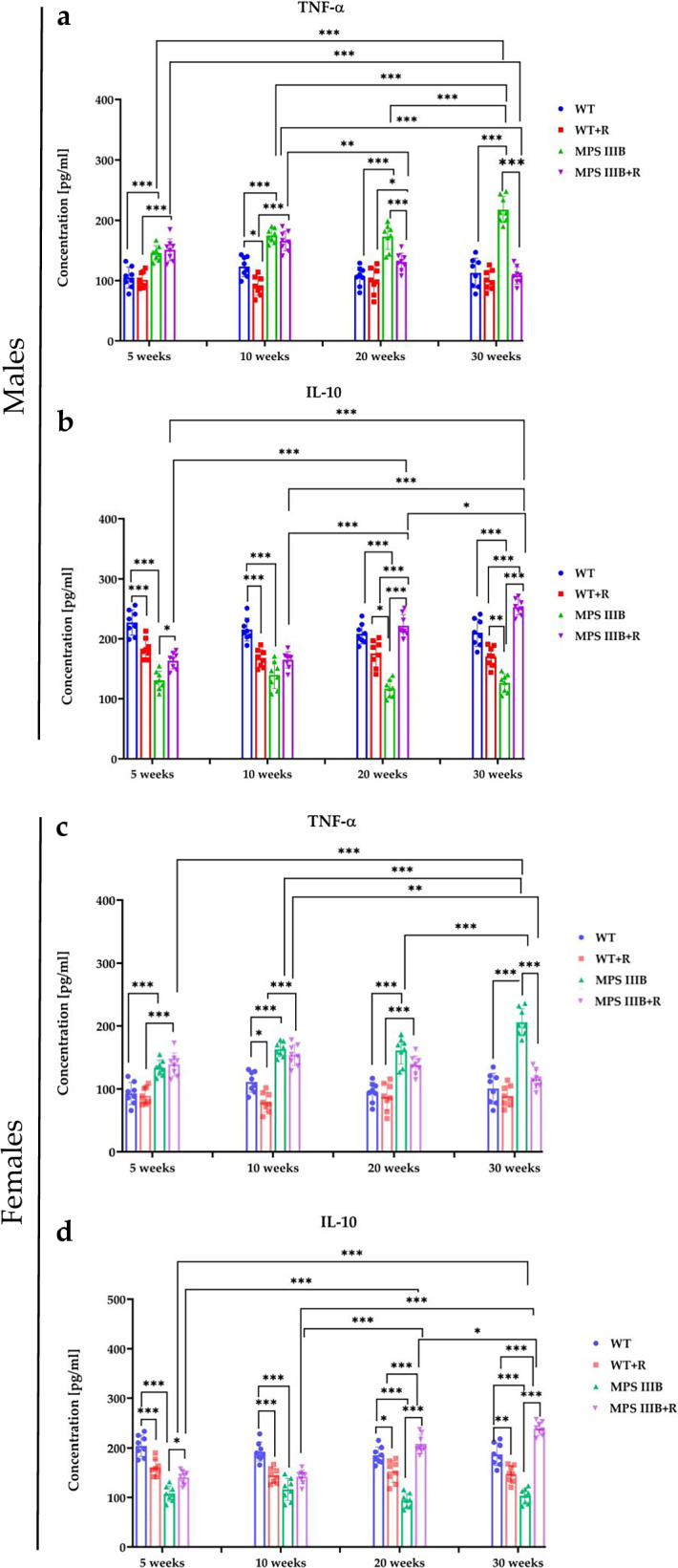
Peripheral cytokine levels at four time points. **a** The plasma TNF level in male mice. **b** The plasma IL-10 level in male mice. **c** The plasma TNF level in female mice. **d** The plasma IL-10 level in female mice. The results are shown as mean values ± s.d. (*n* = 7–8). Statistically significant differences are indicated by asterisks: *P < 0.05, **P < 0.01 and ***P < 0.001.

Since inflammation, and especially neuroinflammation, is one of the crucial secondary effects of HS accumulation^23^, we estimated the levels of major proinflammatory and anti-inflammatory cytokines, TNF and IL-10, respectively, in plasma of MPS IIIB and control mice. In both male and female MPS IIIB mice, we observed a significant increase in the TNF level relative to control animals, while IL-10 was considerably less abundant in the animal model of Sanfilippo disease type B (Fig. 6). The peripheral immune reaction was similar in both male and female MPS IIIB mice. These results indicated intensive inflammatory processes occurring in MPS IIIB mice. The treatment with resveratrol resulted in a reduction in peripheral inflammation, as manifested by a significant decrease in plasma TNF levels, the secretion of which is inhibited by elevated IL-10 levels in MPS IIIB mice. MPS IIIB animals not supplemented with resveratrol showed a significant cytokine imbalance, evident by a nonphysiological decrease in IL-10. The lack of an effective anti-inflammatory response leads to increased inflammation, resulting in the aggravation of different symptoms but also causing neuroinflammation and nonphysiological neuronal activation of brain areas regulating motor and stress responses. Elevated levels of proinflammatory cytokines in the brain lead to adverse modifications of neuronal function, impaired synaptic transmission/plasticity and consequently changes in the activity of various neuronal circuits.

**Fig. 7:**
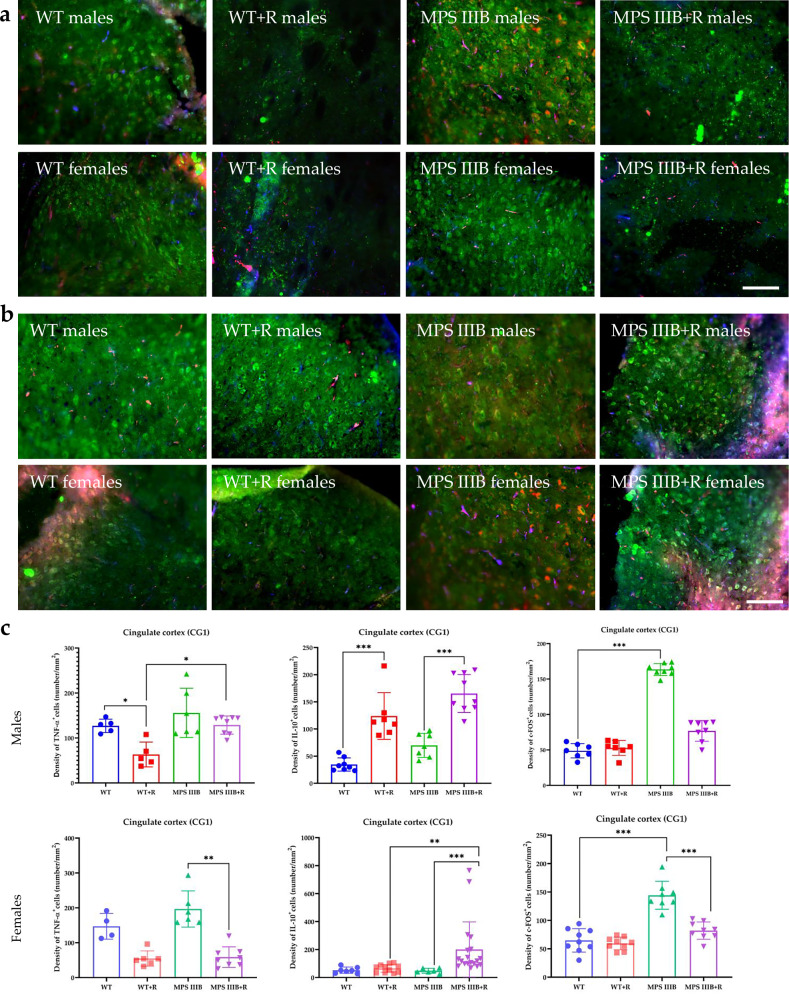
The CG1 immune response. **a** Representative pictures with the density of c-Fos^+^ nuclei (red signal), TNF (green signal) and DAPI (blue signal) labeled neurons (number/1 mm^2^) in the CG1 in both male (top) and female mice (bottom). **b** Representative pictures with the density of c-Fos^+^ nuclei (red signal), IL-10 (green signal) and DAPI (blue signal) labeled neurons (number/1 mm^2^) in the CG1 in both male (top) and female mice (bottom). **c** Statistical analysis of the number of TNF, IL-10 and c-Fos positive cells in both males (top) and females (bottom). Scale bar, 100 μm (lower right corner in **b**) using a fluorescent microscope (PrimoStar from Carl Zeiss MicroImaging GmbH; picture definition 1,024 × 1,024 points; computer program Axio Vision Rel4.8 from Carl Zeiss Imaging System; magnification 20 × 10). The results are shown as mean values ± s.d. (*n* = 7–8). Statistically significant differences are indicated by asterisks: *P < 0.05, **P< 0.01 and ***P < 0.001.

**Fig. 8:**
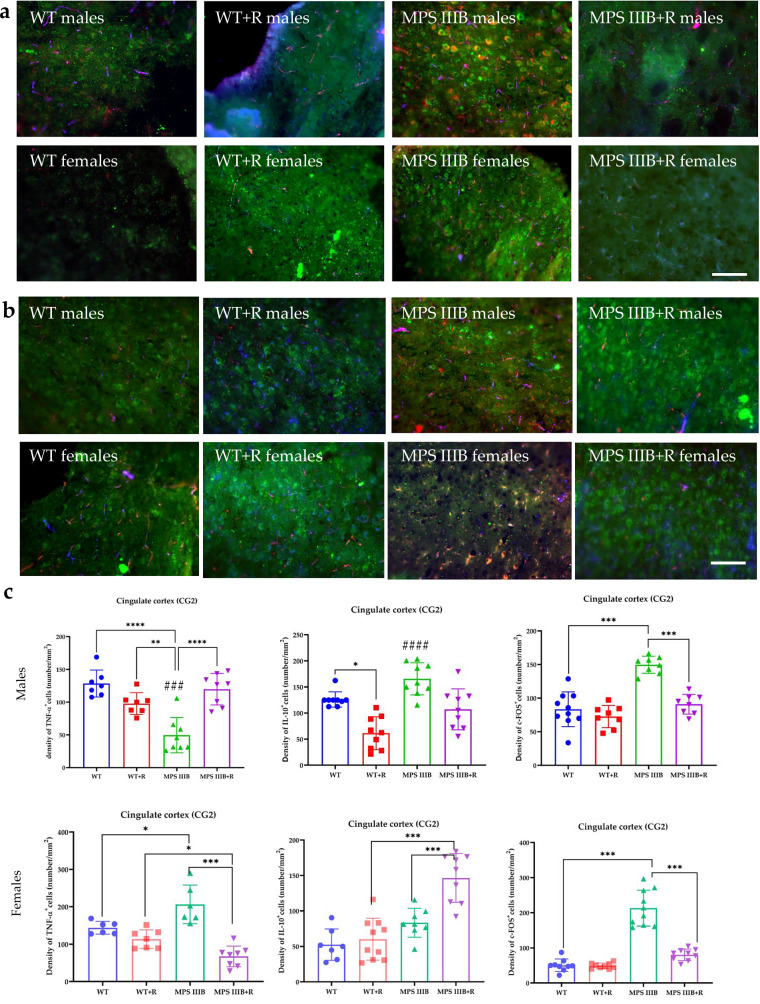
The CG2 immune response. **a** Representative pictures with the density of c-Fos^+^ nuclei (red signal), TNF (green signal) and DAPI (blue signal) labeled neurons (number/1 mm^2^) in the CG2 in both male (top) and female mice (bottom). **b** Representative pictures with the density of c-Fos^+^ nuclei (red signal), IL-10 (green signal) and DAPI (blue signal) labeled neurons (number/1 mm^2^) in the CG2 in both male (top) and female mice (bottom). **c** Statistical analysis of the number of TNF, IL-10 and c-Fos positive cells in both males (top) and females (bottom). Scale bar, 100 μm (lower right corner in **b**) using a fluorescent microscope (fluorescent images as in Fig. 7). The results are shown as mean values ± s.d. (*n* = 7-8). Statistically significant differences are indicated by asterisks: *P < 0.05 and ***P < 0.001. Differences between MPS IIIB males versus MPS IIIB females are indicated with hashtags: ^###^*P* < 0.001 and ^####^*P*< 0.0001.

**Fig. 9:**
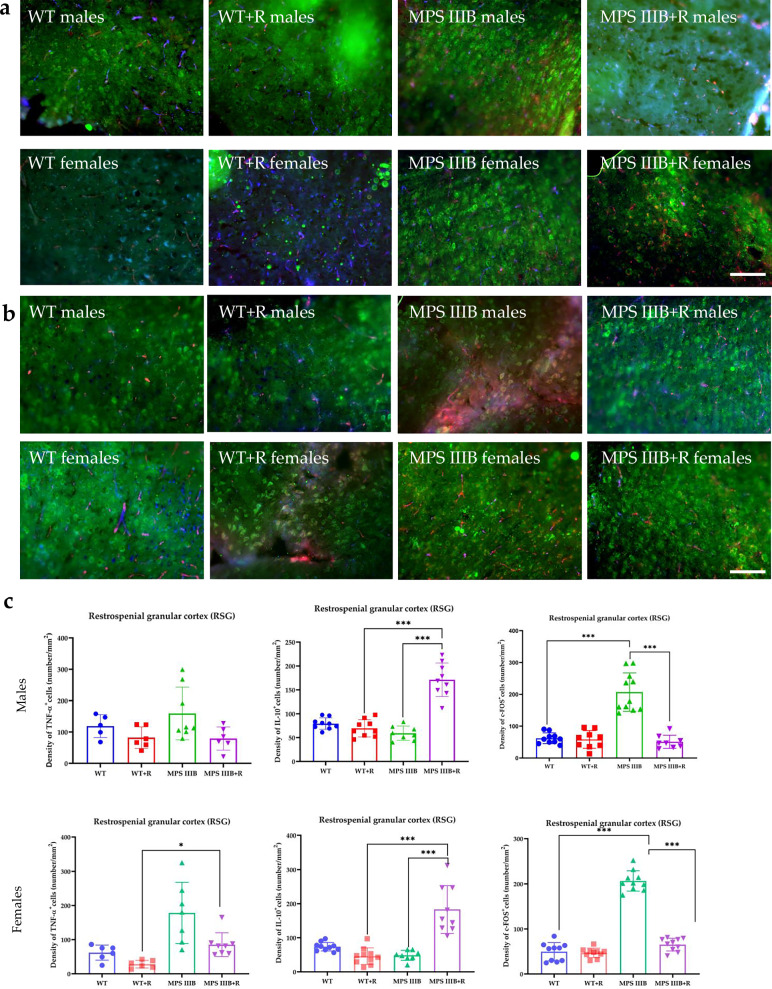
The RSG immune response. **a** Representative pictures with the density of c-Fos^+^ nuclei (red signal), TNF (green signal) and DAPI (blue signal) labeled neurons (number/1 mm^2^) in the RSG in both male (top) and female mice (bottom). **b** Representative pictures with the density of c-Fos^+^ nuclei (red signal), IL-10 (green signal) and DAPI (blue signal) labeled neurons (number/1 mm^2^) in the RSG in both male (top) and female mice (bottom). **c** Statistical analysis of the number of TNF, IL-10 and c-Fos positive cells in both males (top) and females (bottom). Scale bar, 100 μm (lower right corner in **b**) using a fluorescent microscope (fluorescent images as in Fig. 7). The results are shown as mean values ± s.d. (*n* = 7–8). Statistically significant differences are indicated by asterisks: *P < 0.05 and ***P < 0.001.

**Fig. 10:**
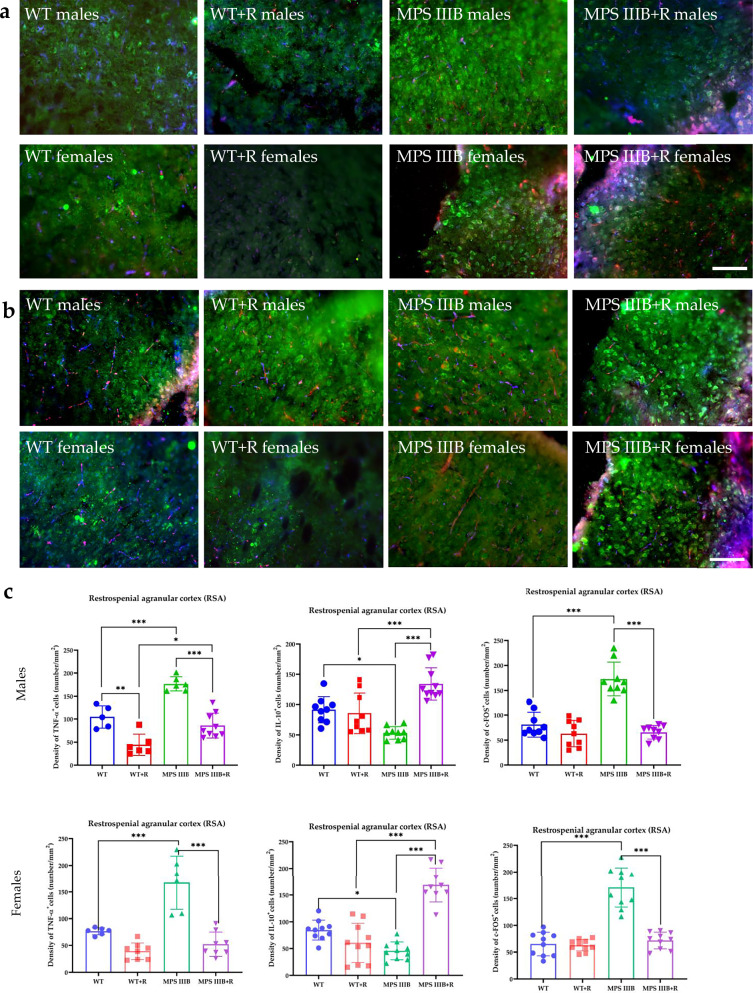
The RSA immune response. **a** Representative pictures with the density of c-Fos^+^ nuclei (red signal), TNF (green signal) and DAPI (blue signal) labeled neurons (number/1 mm^2^) in the RSA in both male (top) and female mice (bottom). **b** Representative pictures with the density of c-Fos^+^ nuclei (red signal), IL-10 (green signal) and DAPI (blue signal) labeled neurons (number/1 mm^2^) in the RSA in both male (top) and female mice (bottom). **c** Statistical analysis of the number of TNF, IL-10 and c-Fos positive cells in both males (top) and females (bottom). Scale bar, 100 μm (lower right corner in **b**) using a fluorescent microscope (fluorescent images as in Fig. 7). The results are shown as mean values ± s.d. (*n* = 7–8). Statistically significant differences are indicated by asterisks: *P < 0.05, **P < 0.01 and ***P < 0.001.

**Fig. 11:**
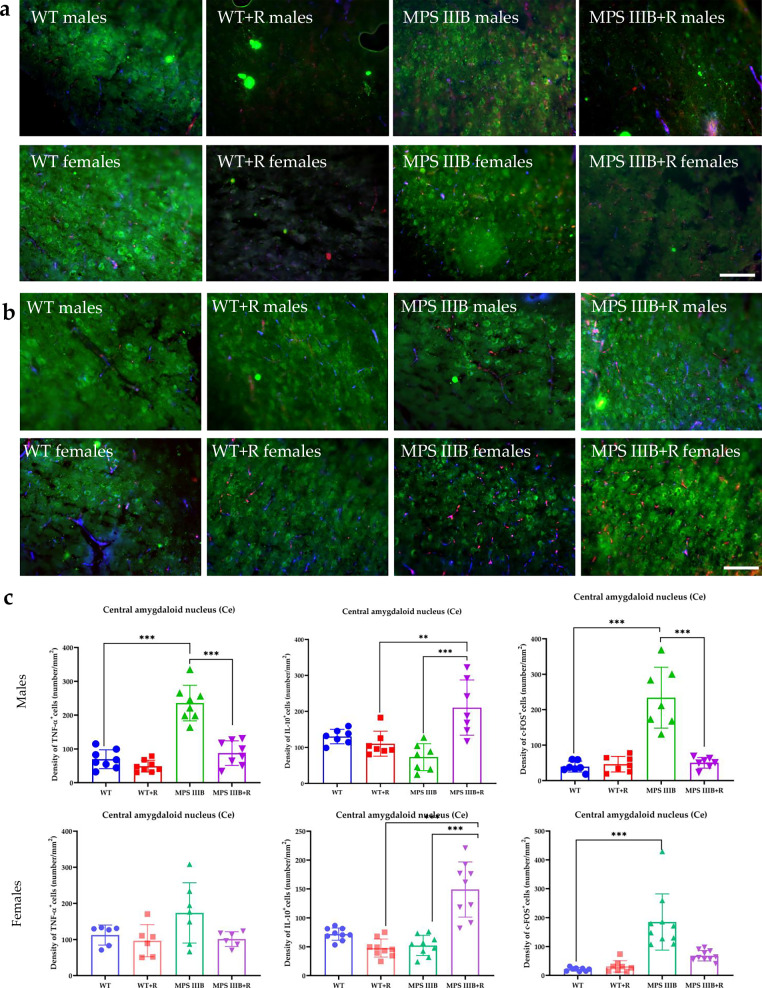
The central nucleus of the amygdala (Ce) immune response. **a** Representative pictures with the density of c-Fos^+^ nuclei (red signal), TNF (green signal) and DAPI (blue signal) labeled neurons (number/1 mm^2^) in the Ce in both male (top) and female mice (bottom). **b** Representative pictures with the density of c-Fos^+^ nuclei (red signal), IL-10 (green signal) and DAPI (blue signal) labeled neurons (number/1 mm^2^) in the Ce in both male (top) and female mice (bottom). **c** Statistical analysis of the number of TNF, IL-10 and c-Fos positive cells in both males (top) and females (bottom). Scale bar, 100 μm (lower right corner in **b**) using a fluorescent microscope (fluorescent images as in Fig. 7). The results are shown as mean values ± s.d. (*n* = 7–8). Statistically significant differences are indicated by asterisks: **P < 0.01 and ***P < 0.001.

**Fig. 12:**
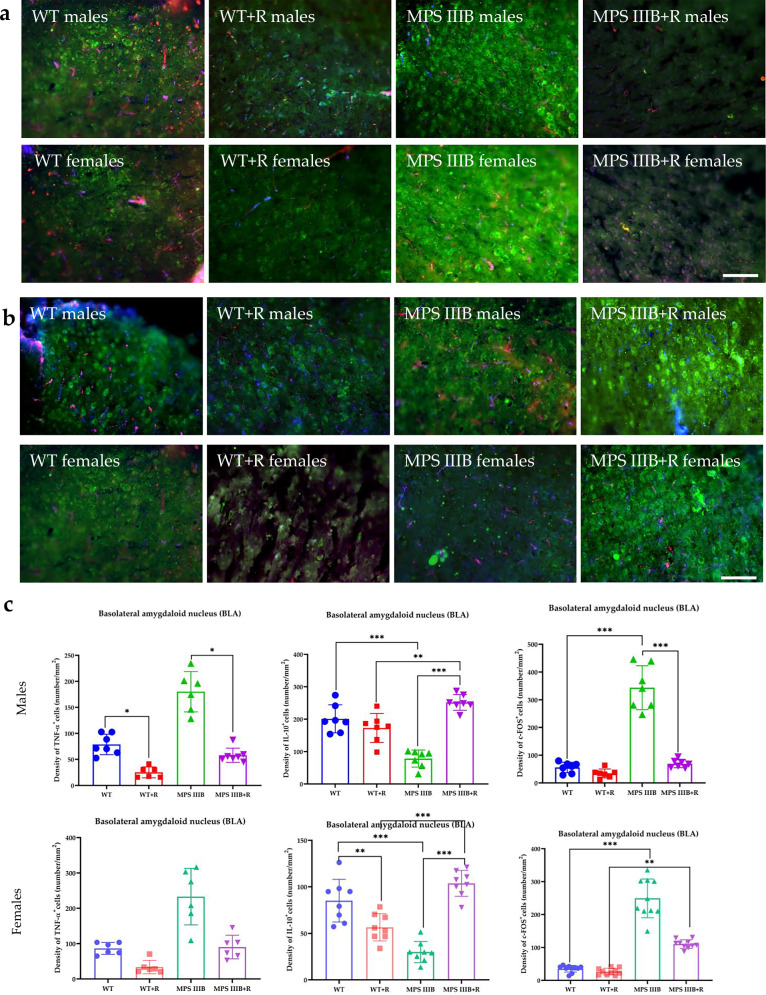
The BLA immune response. **a** Representative pictures with the density of c-Fos^+^ nuclei (red signal), TNF (green signal) and DAPI (blue signal) labeled neurons (number/1 mm^2^) in the BLA in both male (top) and female mice (bottom). **b** Representative pictures with the density of c-Fos^+^ nuclei (red signal), IL-10 (green signal) and DAPI (blue signal) labeled neurons (number/1 mm^2^) in the BLA in both male (top) and female mice (bottom). **c** Statistical analysis of the number of TNF, IL-10 and c-Fos positive cells in both males (top) and females (bottom). Scale bar, 100 μm (lower right corner in **b**) using a fluorescent microscope (fluorescent images as in Fig. 7). The results are shown as mean values ± s.d. (*n* = 7–8). Statistically significant differences are indicated by asterisks: *P < 0.05, **P < 0.01 and ***P < 0.001.

Immunofluorescence microscopy of brain sections from MPS IIIB mice revealed significantly elevated TNF and c-FOS levels, along with abnormally reduced IL-10 abundance in both males and females. These changes were observed in multiple brain regions, including CG1 and CG2 (Figs. 7 and 8), RSG (Fig. 9) and RSA (Fig. 10) and the central (Ce; Fig. 11) and BLA (Fig. 12). These regions are involved in regulating autonomic functions as well as depressive and anxiety-related behaviors. The observed neuroinflammatory and neuronal activation disturbances corresponded closely to behavioral abnormalities detected in open field and anxiety-related tests (Figs. 3–5), including hyperactivity and heightened anxiety. Resveratrol treatment led to region- and sex-specific improvements. In CG1, TNF levels remained unchanged in male MPS IIIB mice but were slightly reduced after treatment, while a significant reduction was observed in females. IL-10 levels increased in both sexes, and elevated c-FOS abundance was reduced, correlating with improved behavior. In CG2 (Fig. 8), female MPS IIIB mice showed a marked increase in TNF compared to WT, which was normalized after resveratrol. TNF was higher in female mice than in male MPS IIIB mice (*P* < 0.0001, *t* = 7.398). While IL-10 levels were higher in males than in females (*P* < 0.00001, *t* = 6.406), resveratrol increased IL-10 significantly in females. The c-FOS level was elevated in both sexes and reduced with treatment. In the RSG region (Fig. 9), TNF levels did not significantly differ between groups, but IL-10 levels increased after resveratrol in both sexes. c-FOS followed a similar pattern as in CG1 and CG2. In the RSA region (Fig. 10), the TNF level was elevated in both male and female MPS IIIB mice and decreased after treatment. The IL-10 level, which was initially reduced, increased following resveratrol. The c-FOS levels were also normalized. In the central amygdala (Ce) (Fig. 11), TNF abundance was significantly elevated only in male MPS IIIB mice and decreased after treatment. IL-10 levels did not differ between groups at baseline but increased post-treatment in both sexes. c-FOS again followed the same trend of increased levels and normalization. Similarly, in the BLA (Fig. 12), resveratrol reduced TNF and c-FOS levels while increasing IL-10 abundance in MPS IIIB mice of both sexes. Therefore, we conclude that resveratrol therapy diminished neuroinflammation without impairing the activity of the neuronal circuits studied, which was evident in the normalization of the behavior of MPS IIIB mice.

### Mechanism of inflammation induction in MPS IIIB and correlation with the autophagy process

**Fig. 13:**
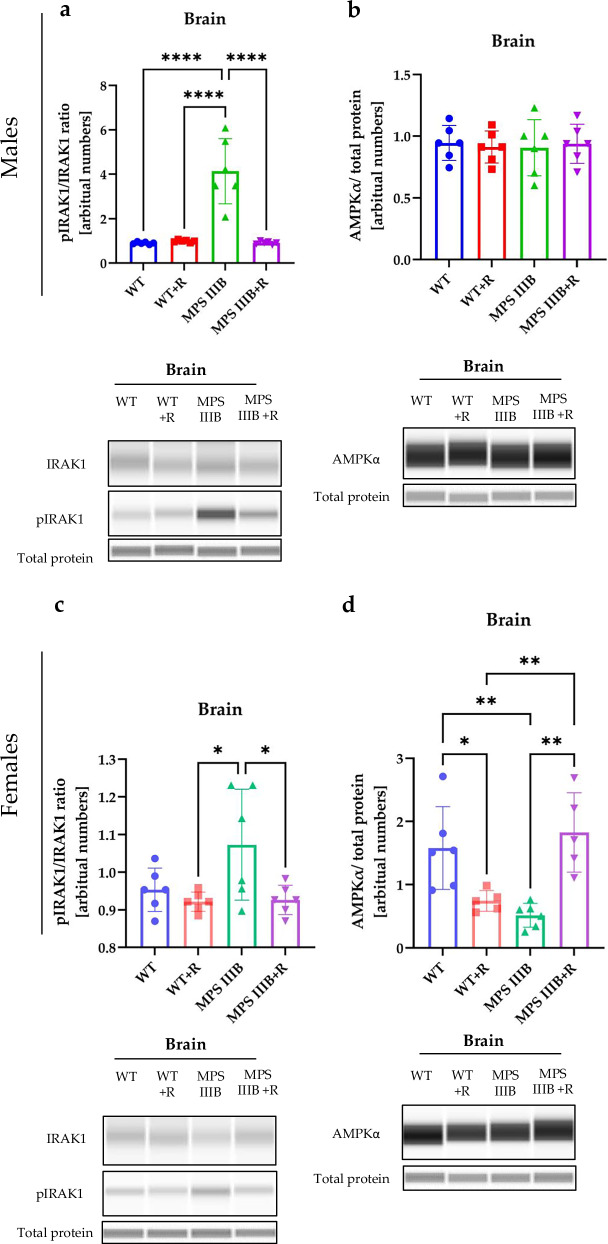
Inflammation induction and correlation with the autophagy process in WT and MPS IIIB mice. **a**, **b** Top: the levels of IRAK1 and pIRAK1 (**a**) and AMPKα (**b**) protein in the brains of male mice shown as either relative to the loading control or the ratio of phosphorylated to total protein. Bottom: representative western blots with statistical analysis in the brains of male mice. **c**, **d** Top: the levels of IRAK1 and pIRAK1 (**c**) and AMPKα (**d**) protein in the brains of female mice shown as either relative to the loading control or the ratio of phosphorylated to total protein. Bottom: representative western blots with statistical analysis in the brains of female mice. The results are shown as mean values ± s.d. (*n* = 6–7). Statistically significant differences are indicated by asterisks: *P< 0.05, **P< 0.01 and ****P < 0.0001.

Neuroinflammation is considered one of the crucial processes involved in neurodegeneration in Sanfilippo disease^23^. Indeed, as indicated above, the levels both peripheral and CNS cytokines were disturbed in the MPS IIIB model (Figs. 6–12). Since the regulation of expression of genes coding for proinflammatory (such as TNF) and anti-inflammatory (such as IL-10) cytokines proceeds through the NF-κB transcription regulator^54^, which is controlled by (among others) the IRAK1 protein^55^ irrespective of the initial signal pathway, we measured the levels of phosphorylated IRAK1 (pIRAK1) relative to total IRAK1 in the brains of the investigated animals. We found that IRAK phosphorylation is significantly increased in MPS IIIB mice (in both males and females) (Fig. 13a, c), which is compatible with the stimulated neuroinflammation process. Previous reports indicate that HS may interact with the TLR4 receptor, a cellular membrane receptor also found on endosomal membranes, where it initiates signal transduction processes, leading to the activation of genes coding for inflammatory proteins^56^. As TLR4-mediated IRAK1 activation induces TNF expression^57^, this could be a potential mechanism for the overactivation of inflammation in MPS IIIB mice.

One of potential activators of the NF-κB stimulation pathway is AMPKα^52^. While levels of this kinase were not significantly different between the brains of MPS IIIB and control males, a considerable decrease in AMPKα abundance was observed in MPS IIIB females relative to WT animals (Fig. 13b, d). These results suggest a difference in the mechanism of neuroinflammation induction between MPS IIIB males and females. Nevertheless, it is worth noting that in MPS IIIB mice (both males and females) receiving resveratrol (150 mg/kg/day since week 8), levels of both factors described in this section (pIRAK and AMPKα) were indistinguishable from those measured in control animals (Fig. 13b, d), pointing to the efficacy of this compound in the normalization of immune processes in the brains of animal models of MPS IIIB.

Since it was reported previously that resveratrol stimulates autophagy in MPS IIIB, which was proposed as the major mechanism in the improvement of the tested disease parameters^33^, in the next stage of this study, we tested whether the autophagy process is impaired in the animal model of Sanfilippo disease type B and investigated the mechanism of resveratrol-mediated stimulation of autophagy in this model. Moreover, since AMPKα is also involved in autophagy activation, a possible interplay between disturbance of the immune response and autophagy was also assessed.

### Autophagy in MPS IIIB mice and effects of resveratrol

**Fig. 14:**
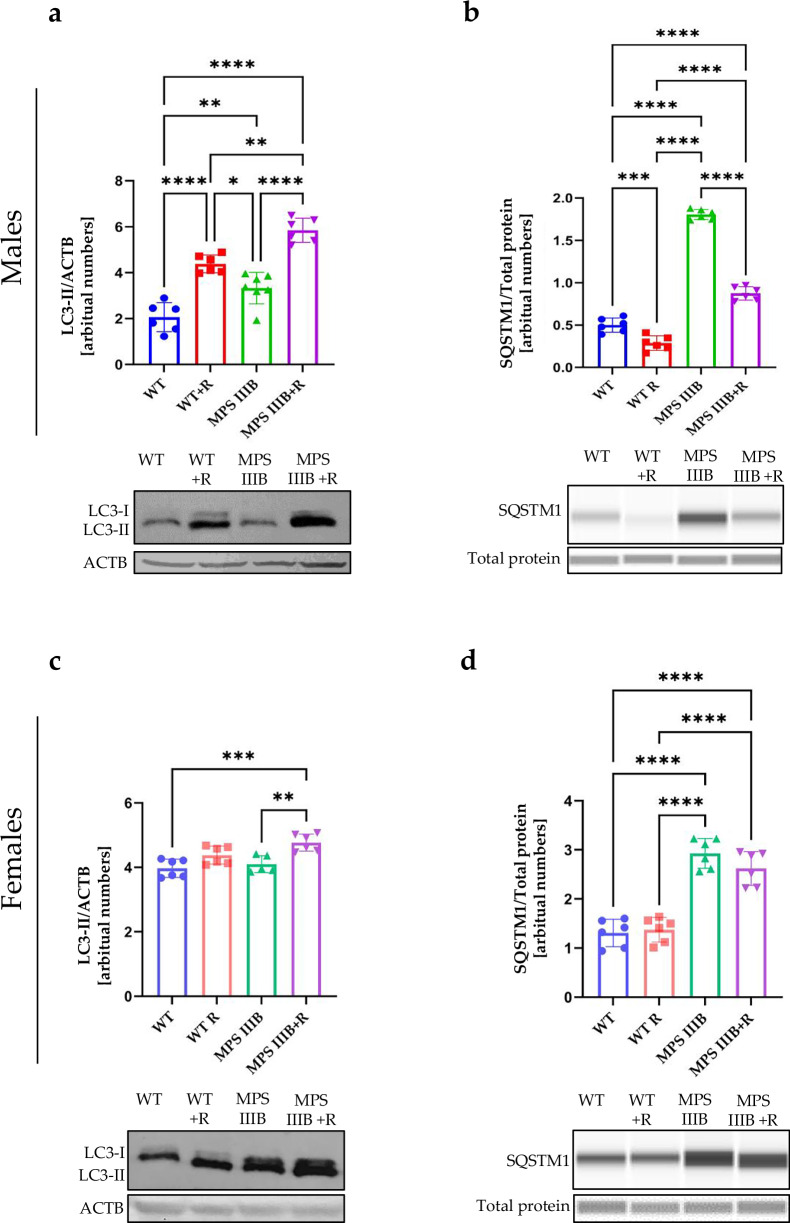
Autophagy activation process in the brain of WT and MPS IIIB mice assessed by LC3-II and SQSTM1 protein levels. **a** Representative western blots of LC3-II protein levels with statistical analysis in the brain of male mice shown as the calculation to loading control. **b** Representative western blots of SQSTM1protein levels with statistical analysis in the brain of male mice shown as the calculation to loading control. **c** Representative western blots of LC3-II protein levels with statistical analysis in the brain of female mice shown as the calculation to loading control. **d** Representative western blots of SQSTM1protein levels with statistical analysis in the brain of female mice shown as the calculation to loading control. The results are shown as mean values ± s.d. (*n* = 6–7). Statistically significant differences are indicated by asterisks: *P < 0.05, **P < 0.01, ***P < 0.001 and ****P < 0.0001.

**Fig. 15:**
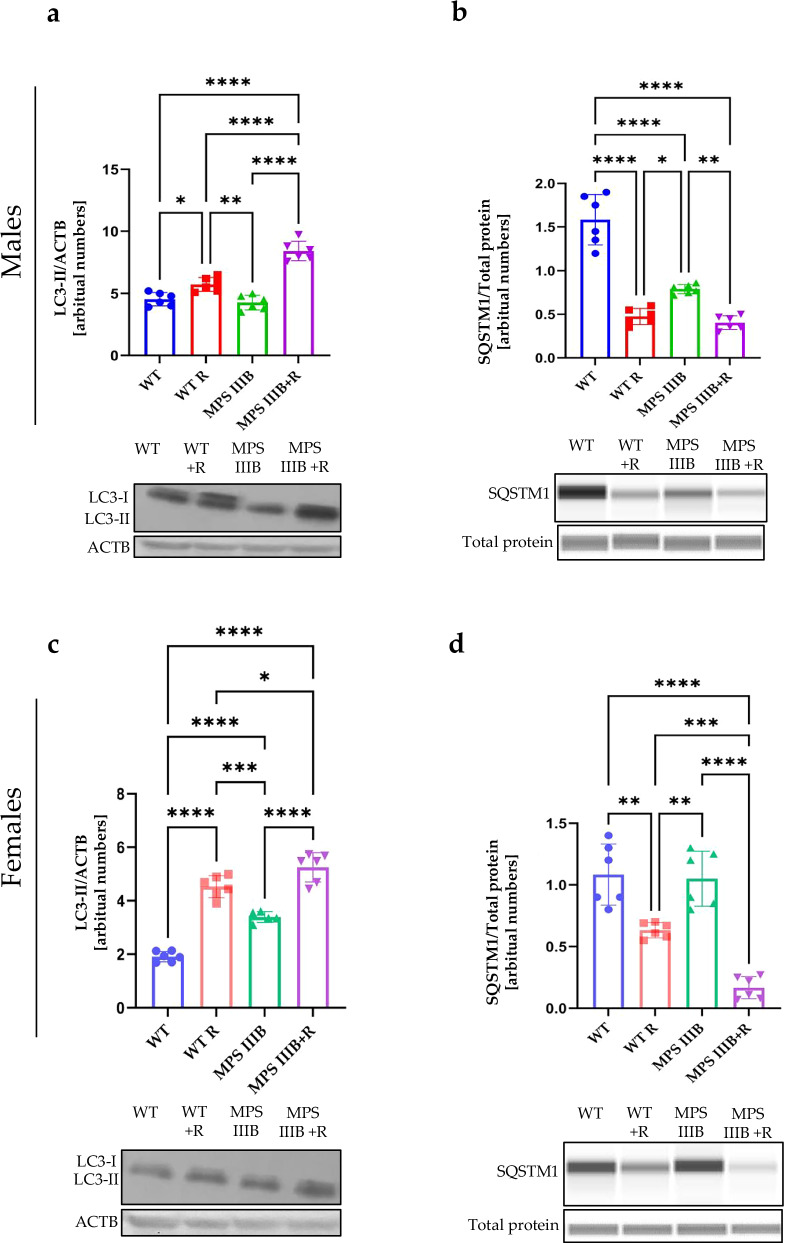
Autophagy activation process in the spleen of WT and MPS IIIB mice as assessed by determining LC3-II and SQSTM1 protein levels. **a** Representative western blots of LC3-II protein levels with statistical analysis in the spleen of male mice shown as the calculation to loading control. **b** Representative western blots of SQSTM1protein levels with statistical analysis in the spleen of male mice shown as the calculation to loading control. **c** Representative western blots of LC3-II protein levels with statistical analysis in the spleen of female mice shown as the calculation to loading control. **d** Representative western blots of SQSTM1protein levels with statistical analysis in the spleen of female mice shown as the calculation to loading control. The results are shown as mean values ± s.d. (*n* = 6–7). Statistically significant differences are indicated by asterisks: *P < 0.05, **P < 0.01, ***P < 0.001 and ****P < 0.0001.

**Fig. 16:**
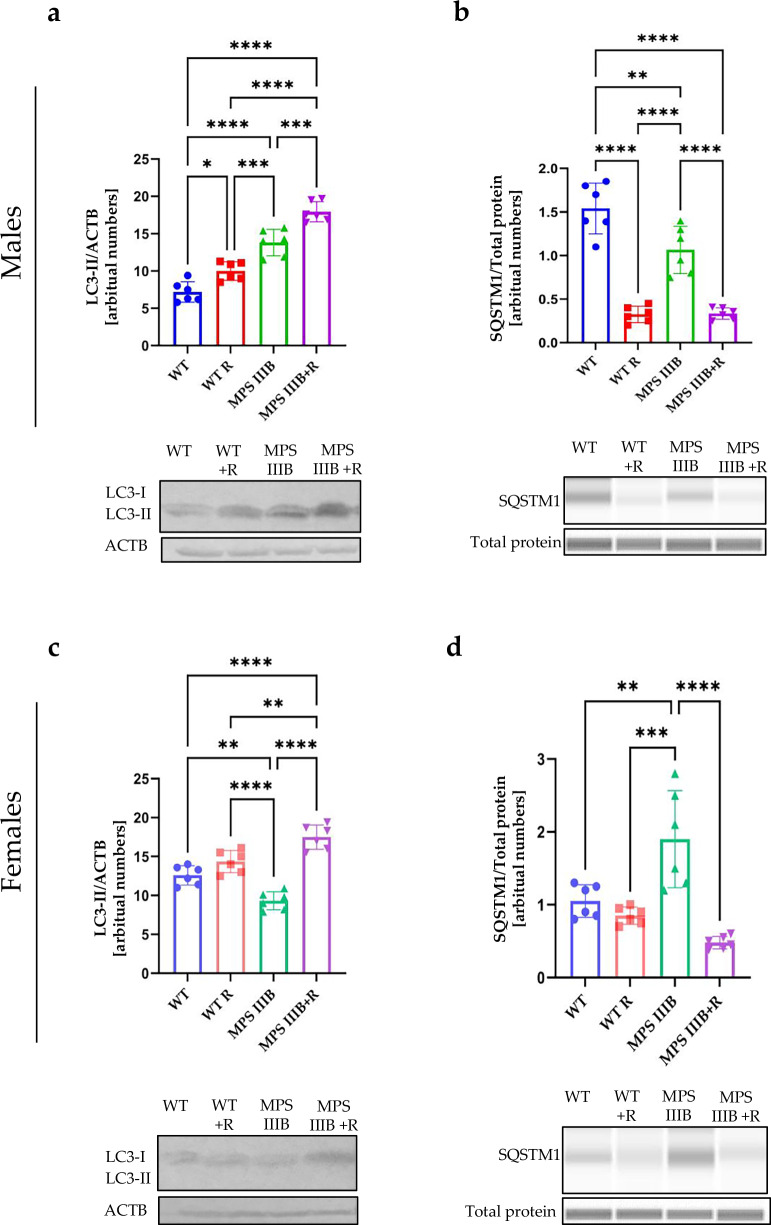
Autophagy activation process in the liver of WT and MPS IIIB mice as assessed by determining LC3-II and SQSTM1 protein levels. **a** Representative western blots of LC3-II protein levels with statistical analysis in the liver of male mice shown as the calculation to loading control. **b** Representative western blots of SQSTM1protein levels with statistical analysis in the liver of male mice shown as the calculation to loading control. **c** Representative western blots of LC3-II protein levels with statistical analysis in the liver of female mice shown as the calculation to loading control. **d** Representative western blots of SQSTM1protein levels with statistical analysis in the liver of female mice shown as the calculation to loading control. The results are shown as mean values ± s.d. (*n* = 6–7). Statistically significant differences are indicated by asterisks: *P < 0.05, **P < 0.01, ***P < 0.001 and ****P < 0.0001.

To test whether autophagy is affected in MPS IIIB mice (males and females), we determined the levels of the LC3-II protein, a marker of autophagy, in different organs (brain, spleen and liver). This protein was more abundant in the brain and liver (Figs. 14a and16a), but not in the spleen (Fig. 15a), of MPS IIIB males than in control males. However, in females, no significant differences could be detected in the levels of LC3-II in the brains of MPS IIIB and healthy animals (Fig. 14c), while the investigated protein was more abundant in the spleen (Fig. 15c) and less abundant in the liver of MPS IIIB mice relative to controls (Fig. 16c). Therefore, there were differences between MPS III males and females in the steady-state levels of this autophagy marker (LC3-II), and the variability in this marker was also evident between organs. In some organs, autophagy stimulation was probably an indication of the cellular stress response, which was evidently not efficient enough to eliminate the storage of HS. Nevertheless, it is striking that treatment with resveratrol resulted in a significant increase in LC3-II abundancy in all experimental systems used, leading to considerably higher levels of this autophagy marker than in untreated animals (Figs. 14a,c, 15a,c and 16a, c).

Another autophagy marked is the SQSTM1/p62 protein^58^; however, in this case, elevated levels of this protein suggest the inhibition of autophagy flux and an inability to degrade this marker. The levels of SQSTM1 were significantly increased in the brains of MPS IIIB males and females relative to control animals (Fig. 14b, d), suggesting impaired autophagy in the brain. In contrast to the brain (Fig. 14b), SQSTM1 was less abundant in the spleen (Fig. 15b) and liver (Fig. 16b) of MPS IIIB males relative to controls, while in MPS IIIB females, levels of this protein were either unchanged or elevated in the spleen (Fig. 15d) and liver (Fig. 16d), respectively. Again, differences between sexes and organs were evident in the autophagy process occurring in MPS IIIB mice. However, resveratrol administration caused an evident decrease in SQSTM1 levels in all tested organs in both sexes, indicating effective induction of the autophagy process.

**Fig. 17:**
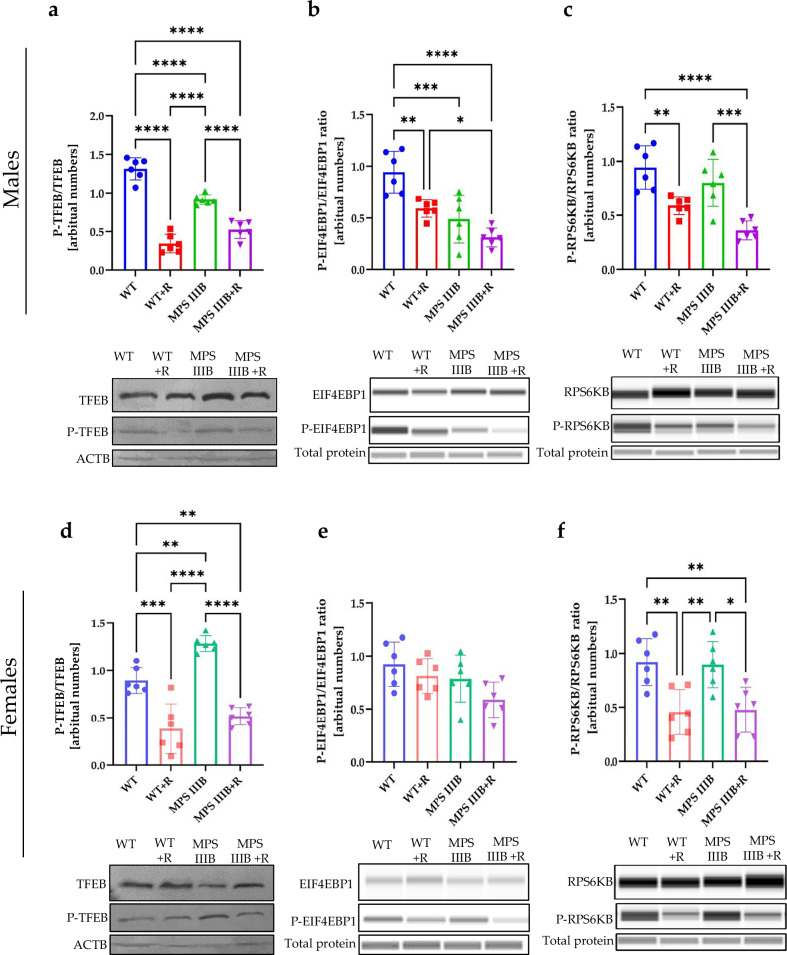
mTOR substrates TFEB, EIF4EBP1 and RPS6KB in the brain of WT and MPS IIIB mice. **a** Representative western blots of P-TFEB and TFEB protein levels in the brain of male mice shown as the ratio of phosphorylated to total protein. **b** Representative western blots of P-EIF4EBP1 and EIF4EBP1 protein levels in the brain of male mice shown as ratio of phosphorylated to total protein. **c** Representative western blots of P-RPS6KB and RPS6KB protein levels in the brain of male mice shown as the ratio of phosphorylated to total protein. **d** Representative western blots of P-TFEB and TFEB protein levels in the brain of female mice shown as the ratio of phosphorylated to total protein. **e** Representative western blots of P-EIF4EBP1 and EIF4EBP1 protein levels in the brain of female mice shown as the ratio of phosphorylated to total protein. **f** Representative western blots of P-RPS6KB and RPS6KB protein levels in the brain of female mice shown as the ratio of phosphorylated to total protein. The results are shown as mean values ± s.d. (*n* = 6–7). Statistically significant differences are indicated by asterisks: *P< 0.05, **P < 0.01, ***P < 0.001 and ****P < 0.0001.

**Fig. 18:**
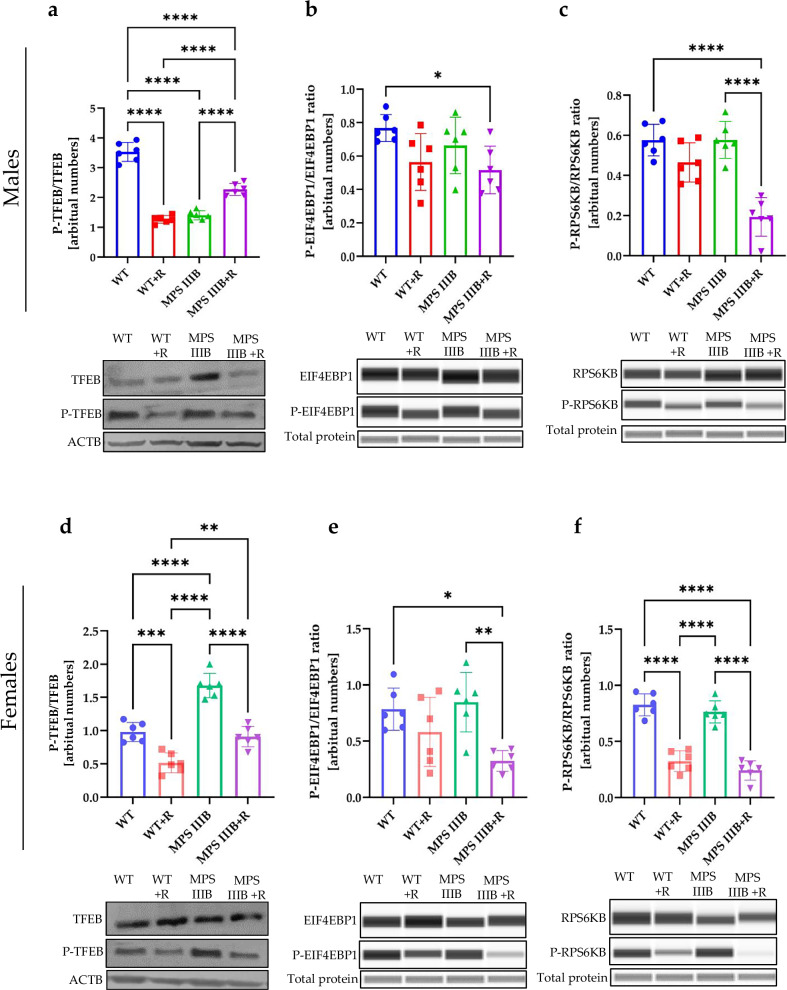
mTOR substrates TFEB, EIF4EBP1 and RPS6KB in the spleen of WT and MPS IIIB mice. **a** Representative western blots of P-TFEB and TFEB protein levels in the spleen of male mice shown as the ratio of phosphorylated to total protein. **b** Representative western blots of P-EIF4EBP1 and EIF4EBP1 protein levels in the spleen of male mice shown as ratio of phosphorylated to total protein. **c** Representative western blots of P-RPS6KB and RPS6KB protein levels in the spleen of male mice shown as the ratio of phosphorylated to total protein. **d** Representative western blots of P-TFEB and TFEB protein levels in the spleen of female mice shown as the ratio of phosphorylated to total protein. **e** Representative western blots of P-EIF4EBP1 and EIF4EBP1 protein levels in the spleen of female mice shown as the ratio of phosphorylated to total protein. **f** Representative western blots of P-RPS6KB and RPS6KB protein levels in the spleen of female mice shown as the ratio of phosphorylated to total protein. The results are shown as mean values ± s.d. (*n* = 6–7). Statistically significant differences are indicated by asterisks: *P < 0.05, **P < 0.01, ***P < 0.001 and ****P < 0.0001.

**Fig. 19:**
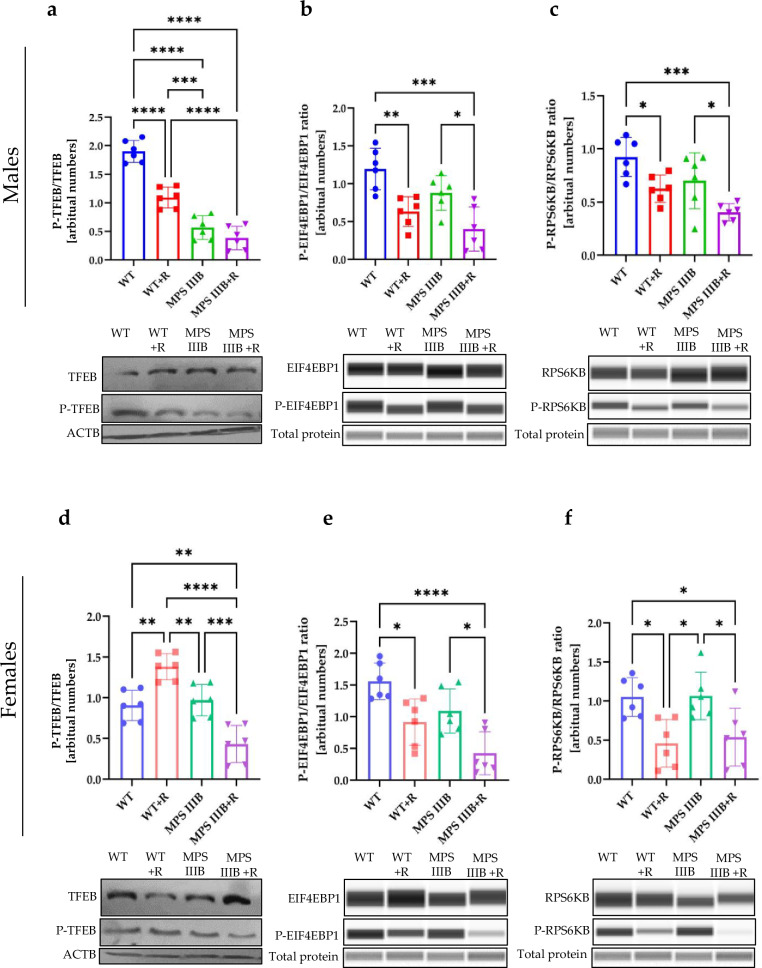
mTOR substrates TFEB, EIF4EBP1 and RPS6KB in the liver of WT and MPS IIIB mice. **a** Representative western blots of P-TFEB and TFEB protein levels in the liver of male mice shown as the ratio of phosphorylated to total protein. **b** Representative western blots of P-EIF4EBP1 and EIF4EBP1 protein levels in the liver of male mice shown as ratio of phosphorylated to total protein. **c** Representative western blots of P-RPS6KB and RPS6KB protein levels in the liver of male mice shown as the ratio of phosphorylated to total protein. **d** Representative western blots of P-TFEB and TFEB protein levels in the liver of female mice shown as the ratio of phosphorylated to total protein. **e** Representative western blots of P-EIF4EBP1 and EIF4EBP1 protein levels in the liver of female mice shown as the ratio of phosphorylated to total protein. **f** Representative western blots of P-RPS6KB and RPS6KB protein levels in the liver of female mice shown as the ratio of phosphorylated to total protein. The results are shown as mean values ± s.d. (*n* = 6–7). Statistically significant differences are indicated by asterisks: *P < 0.05, **P < 0.01, ***P < 0.001 and ****P < 0.0001.

There are several pathways of autophagy stimulation, divided into two major groups: mTOR dependent and mTOR independent^58,59^. The mTOR kinase phosphorylates various factors, thus impairing autophagy induction. Therefore, we measured levels of phosphorylation of mTOR substrates and the effects of resveratrol on this parameter. Phosphorylation of the transcription factor EB (TFEB) prevents its translocation from cytoplasm to the nucleus, leading to inhibition of lysosome biogenesis, thus indirectly impairing autophagy^58^. We detected decreased levels of the phosphorylated TFEB (P-TFEB) form relative to total TFEB in brains (Fig. 17a), spleen (Fig. 18a) and liver (Fig. 19a) of MPS IIIB males compared to control mice, which is compatible with stimulated autophagy initiation, as estimated by determining elevated levels of LC3-II (Figs. 14a, 15a and16a) and SQSTM (Figs. 14b, 15b and 16b). However, in MPS IIIB females, the phosphorylation of TFEB was either increased (in the brain (Fig. 17d) and spleen (Fig. 18d)) or unaffected (in the liver (Fig. 19d)), indicating significant differences between the processes occurring in the cells of MPS IIIB mice of different sexes. Nonetheless, in all groups treated with resveratrol, the levels of P-TFEB were low, showing that the mTOR-dependent pathway of autophagy stimulation is induced. These results were further confirmed in a cellular model, where resveratrol treatment induced the translocation of TFEB from the cytoplasm to the nucleus (Supplementary Fig. 2). This conclusion could be corroborated by the investigation of another substrate for mTOR, the EIF4EBP1 and RPS6KB proteins. Indeed, phosphorylated forms of these proteins (P-EIF4EBP1 and P-RPS6KB) were significantly less abundant after treatment with resveratrol in all experimental systems tested (Figs. 17–19b, c, e, f).

**Fig. 20:**
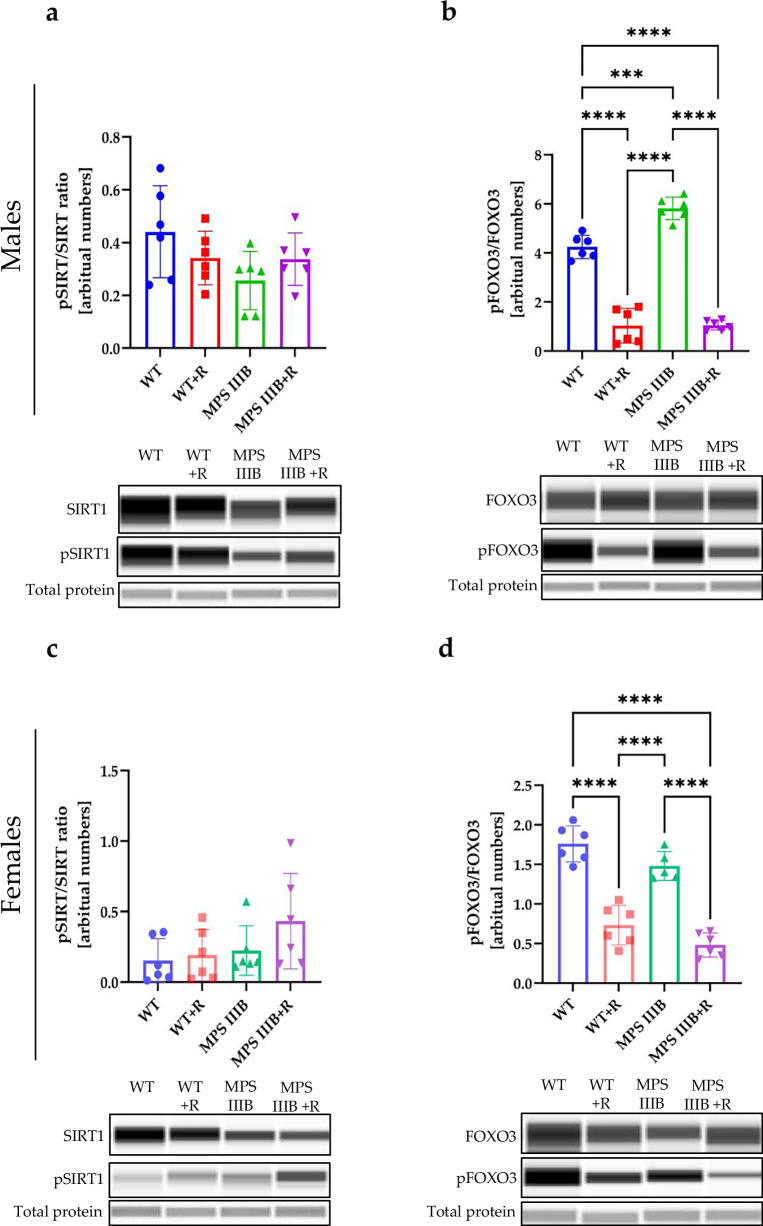
Sirtuin and FOXO activation by resveratrol in the brain of WT and MPS IIIB mice. **a** Representative western blots of P-SIRT1 and SIRT1 protein levels in the brain of male mice shown as the ratio of phosphorylated to total protein. **b** Representative western blots of P-FOXO3 and FOXO3 protein levels in the brain of male mice shown as the ratio of phosphorylated to total protein. **c** Representative western blots of P-SIRT1 and SIRT1 protein levels in the brain of female mice shown as the ratio of phosphorylated to total protein. **d** Representative western blots of P-FOXO3 and FOXO3 protein levels in the brain of female mice shown as the ratio of phosphorylated to total protein. The results are shown as mean values ± s.d. (*n* = 6–7). Statistically significant differences are indicated by asterisks: ***P < 0.001 and ****P < 0.0001.

**Fig. 21:**
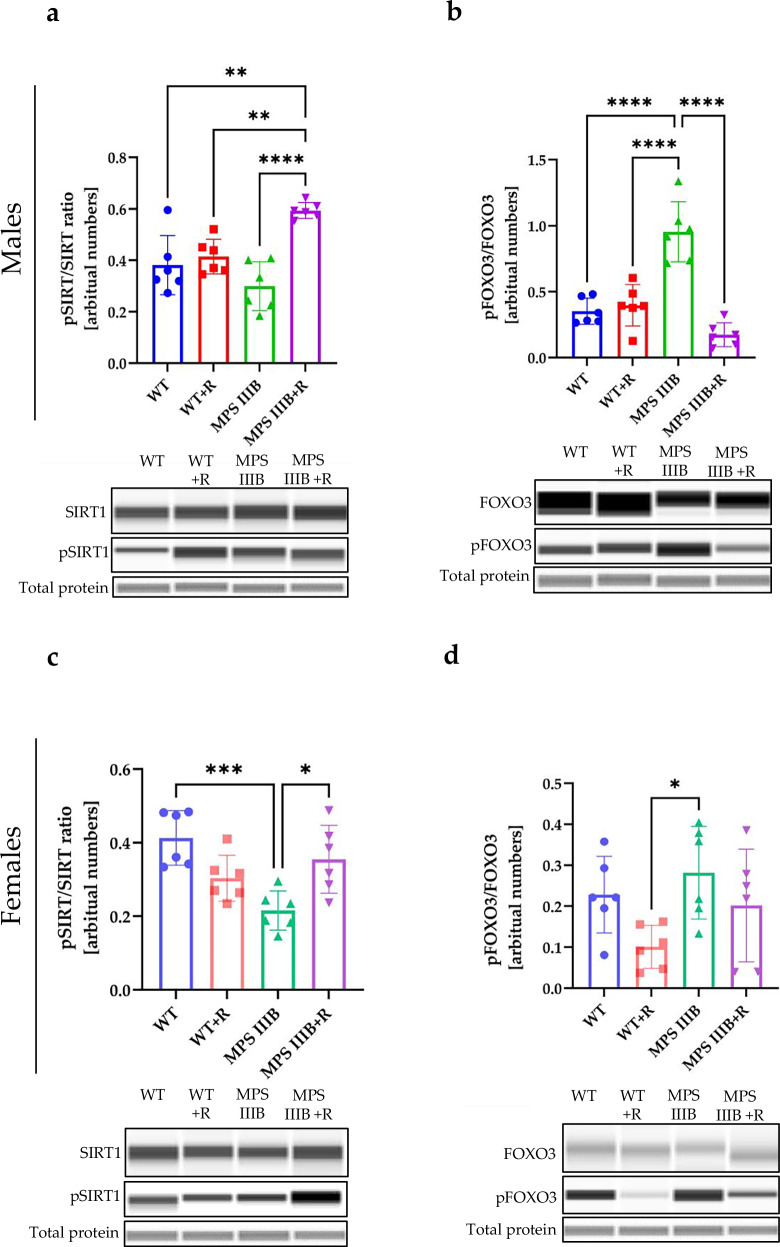
Sirtuin and FOXO activation by resveratrol in the spleen of WT and MPS IIIB mice. **a** Representative western blots of P-SIRT1 and SIRT1 protein levels in the spleen of male mice shown as the ratio of phosphorylated to total protein. **b** Representative western blots of P-FOXO3 and FOXO3 protein levels in the spleen of male mice shown as the ratio of phosphorylated to total protein. **c** Representative western blots of P-SIRT1 and SIRT1 protein levels in the spleen of female mice shown as the ratio of phosphorylated to total protein. **d** Representative western blots of P-FOXO3 and FOXO3 protein levels in the spleen of female mice shown as the ratio of phosphorylated to total protein. The results are shown as mean values ± s.d. (*n* = 6–7). Statistically significant differences are indicated by asterisks: *P < 0.05, **P < 0.01, ***P < 0.001 and ****P < 0.0001.

**Fig. 22:**
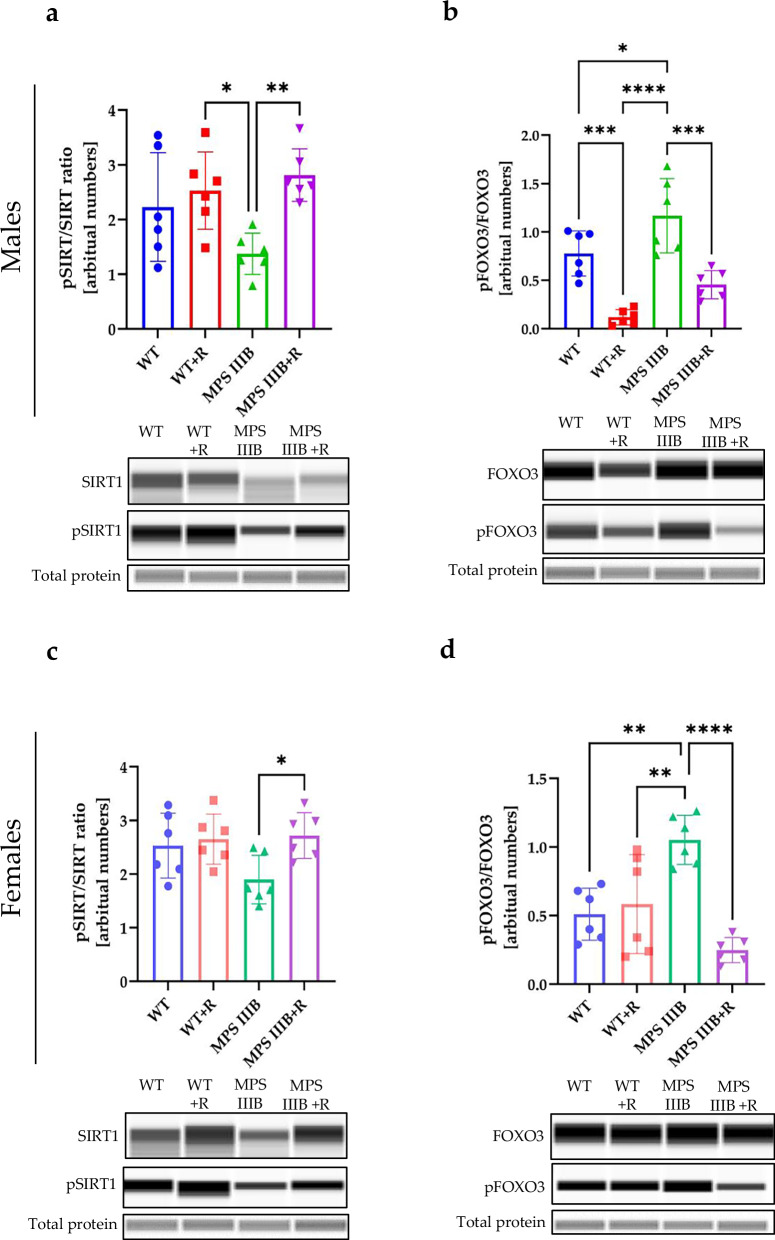
Sirtuin and FOXO activation by resveratrol in the liver of WT and MPS IIIB mice. **a** Representative western blots of P-SIRT1 and SIRT1 protein levels in the liver of male mice shown as the ratio of phosphorylated to total protein. **b** Representative western blots of P-FOXO3 and FOXO3 protein levels in the liver of male mice shown as the ratio of phosphorylated to total protein. **c** Representative western blots of P-SIRT1 and SIRT1 protein levels in the liver of female mice shown as the ratio of phosphorylated to total protein. **d** Representative western blots of P-FOXO3 and FOXO3 protein levels in the liver of female mice shown as the ratio of phosphorylated to total protein. The results are shown as mean values ± s.d. (*n* = 6–7). Statistically significant differences are indicated by asterisks: *P < 0.05, **P < 0.01, ***P < 0.001 and ****P < 0.0001.

Beclin 1 is another protein involved in the autophagy regulation. However, we did not detect any significant differences in levels of Beclin 1 between MPS IIIB and control mice in any tested organ or sex, with or without treatment with resveratrol (Supplementary Fig. 3). It has also been reported that AMPK regulates sirtuin 1 (SIRT1), which influences FOXO3 activity^60^, and dephosphorylation of FOXO3 can lead to autophagy stimulation^47^. Moreover, resveratrol has been suggested to activate SIRT1, which can also result in decreased production of proinflammatory cytokines such as TNF^60^. Therefore, we measured levels of phosphorylated SIRT1 (P-SIRT1) relative to total SIRT is the brain, spleen and liver (Figs. 20–22) of MPS IIIB and control mice and found that administration of resveratrol resulted in a significant increase in the abundance of pSIRT1 in the spleen (Fig. 21a, c) and liver (Fig. 22a, c) of both MPS IIIB males and females. The average pSIRT1/SIRT1 ratios were higher in the brains of resveratrol-treated mice than in untreated animals, but the differences did not reach a statistical significance (Fig. 20a, c). Importantly, the ratio of phosphorylated FOXO3 (P-FOXO3) to total FOXO3 was significantly lower in MPS IIIB mice (both males and females) in the groups treated with resveratrol relative to untreated (Figs. 20b,d, 21b and 22b, d); this was true for all organs except the spleen in MPS IIIB females (Fig. 21d). There were higher levels of P-FOXO3 in untreated MPS IIIB males in all tested organs and in the liver of untreated MPS IIIB females in comparison to WT mice, suggesting that there may be an autophagy block on this pathway in MPS IIIB mice. Moreover, these results strongly suggested that autophagy can be stimulated by resveratrol in different organs of MPS IIIB mice through the FOXO3-dependent pathway, but also indicated that normalization of the immune response, due to reduction of the inflammatory process, may proceed in these animals, at least partially, through resveratrol-induced stimulation of SIRT1 phosphorylation.

### In vitro confirmation of the mechanism of resveratrol-stimulated autophagy in MPS IIIB

**Fig. 23:**
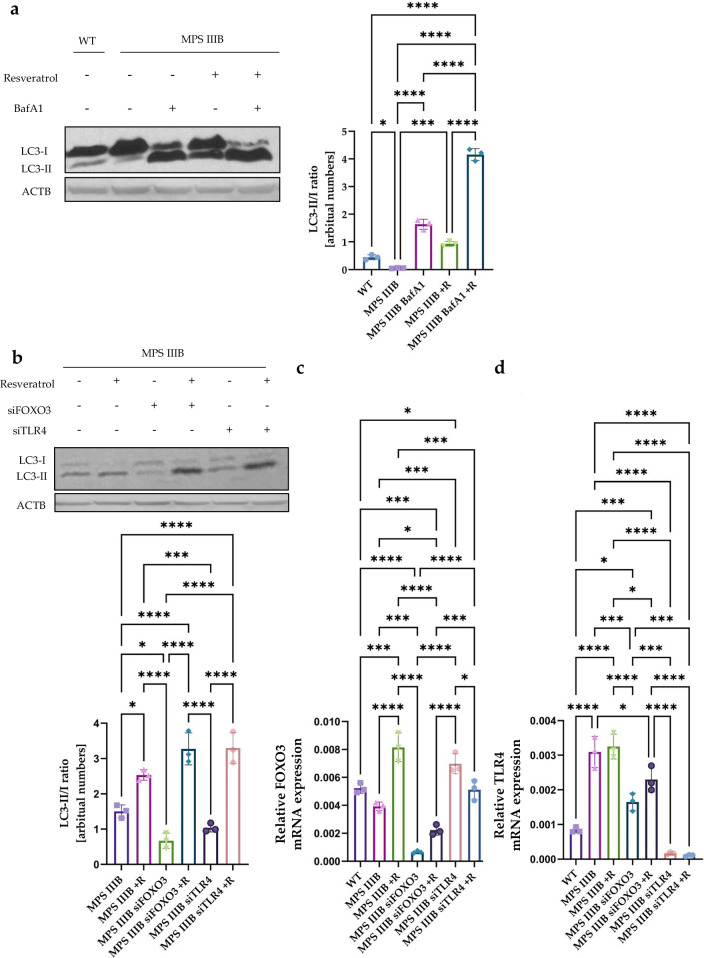
Resveratrol activates autophagy in MPS IIIB. **a** Representative western blots of LC3-II protein levels with statistical analysis, shown as ratio of LC3-II to LC3-I. **b** Representative western blots of LC3-II protein levels with statistical analysis, shown as the ratio of LC3-II to LC3-I. **c** Relative *FOXO3* mRNA expression. **d** Relative TLR4 mRNA expression. The results are shown as mean values ± s.d. (*n* = 3). Statistically significant differences are indicated by asterisks: *P < 0.05, ***P < 0.001 and ****P < 0.0001.

To confirm the FOXO3-dependent mechanism of resveratrol-mediated stimulation of autophagy in MPS IIIB, we performed in vitro experiments using a mouse fibroblast line derived from the animals used as models in the experiments described previously. First, although we observed elevated levels of LC3-II in the brain, spleen and liver of MPS IIIB mice treated with resveratrol relative to untreated animals (Fig. 14), an increase in abundance of this protein can indicate either activation of autophagy or inhibition of the autophagy flux^58^. To distinguish between these two possibilities, we used cultures of mouse MPS IIIB fibroblasts treated with bafilomycin A, a strong inhibitor of the autophagy flux. As expected, treatment with this compound resulted in a significant increase in LC3-II levels (due to inhibition of LC3-II degradation), similar to the results obtained after treatment with resveratrol (Fig. 23a). If resveratrol inhibited autophagy flux (like bafilomycin A), we should observe no considerable differences in LC3-II levels between cells treated with bafilomycin A alone and those treated with both compounds. However, if resveratrol were able to activate autophagy, reflected by enhanced production of form II of the LC3 protein, then levels of LC3-II should be higher in cells treated with both bafilomycin A and resveratrol than in cells treated with bafilomycin alone. In fact, we observed the latter situation (Fig. 23a), corroborating the conclusion that resveratrol stimulates autophagy in MPS IIIB cells.

To test whether FOXO3-dependent regulation is involved in resveratrol-mediated autophagy stimulation, we have investigated the effects of silencing the expression of the *FOXO3* gene using specific siRNA molecules. In addition, to test whether there is a correlation between autophagy and the immune response, we silenced *TLR4* gene expression using specific siRNA molecules (Fig. 23b). Our results showed that silencing the *FOXO3* gene resulted in decreased LC3-II protein levels in MPS IIIB cells in comparison to untreated cells, confirming that autophagy is FOXO3 dependent in MPS IIIB cells. Interestingly, silencing of the *TLR4* gene also decreased autophagy, suggesting that this immune system-related receptor also influences the autophagy process. What is more, resveratrol treatment resulted in activation of autophagy in all treated groups (MPS IIIB and both the silenced genes) (Fig. 23b). Furthermore, silencing of the *SIRT1* gene using specific siRNA did not abolish the effects of resveratrol as FOXO3 translocation to the nucleus was still observed. Consistently, resveratrol treatment continued to activate autophagy under siSIRT1 conditions, as evidenced by an increased LC3-II/I ratio (Supplementary Figs. 4 and 5). Importantly, experiments using scrambled siRNA (siSCR) as a negative control demonstrated that these effects were specific to the corresponding gene-targeting siRNAs.

**Fig. 24:**
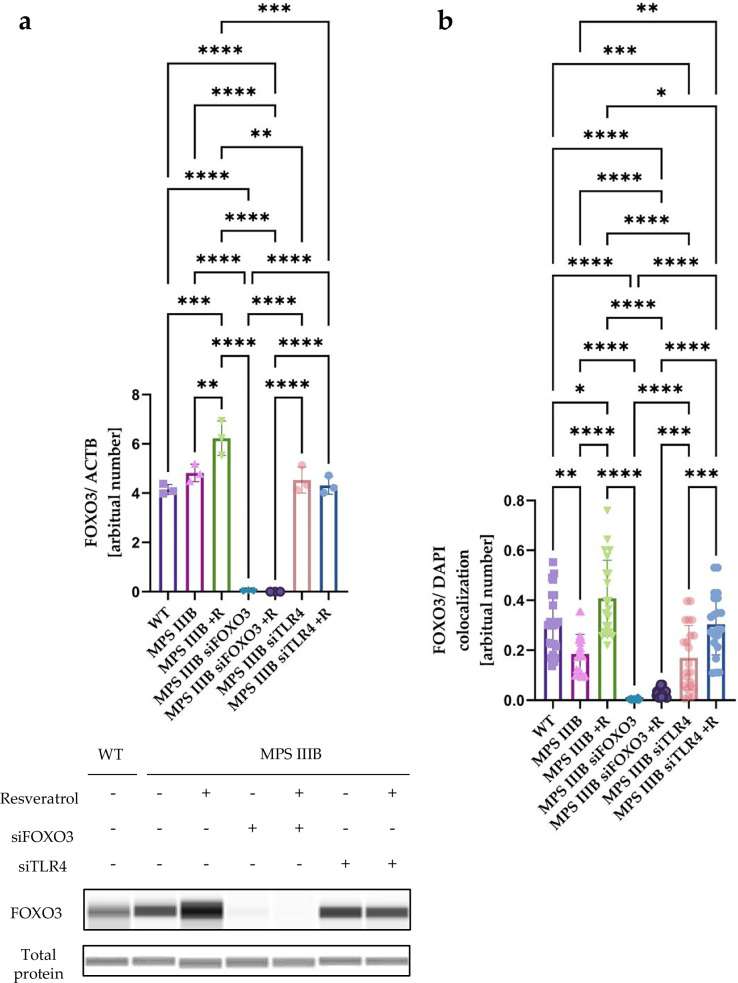
Resveratrol activates autophagy through FOXO3 in MPS IIIB. **a** Representative western blots of FOXO3 protein levels with statistical analysis, shown as the calculation to loading control. **b** Statistical analysis of the Pearson’s correlation coefficient of nucleus fluorescence. The results are shown as mean values ± s.d. (*n* = 3). Statistically significant differences are indicated by asterisks: *P < 0.05, ***P < 0.001 and ****P < 0.0001.

To confirm that the silencing of the genes was effective, RT–qPCR experiments and western blotting were conducted to measure both mRNA and protein levels (Fig. 23c,d and Supplementary Figs. 5 and 6). Indeed, the expression of both *FOXO3* and *TLR4* genes was effectively silenced (Fig. 23c,d and Supplementary Figs. 5 and 6). After treatment with resveratrol, levels of the *FOXO3* gene-derived mRNA were increased in MPS IIIB cells. It is interesting that after silencing the *TLR4* gene, *FOXO3*-derived mRNA levels increased, suggesting that there is a correlation between FOXO3 and the TLR4 receptor (Fig. 23c). On the other hand, silencing of the *FOXO3* gene resulted in decreased expression of the *TLR4* gene (Fig. 23d). Resveratrol treatment did not influence expression of the *TLR4* gene in MPS IIIB mice. However, *TLR4* expression was increased in MPS IIIB cells in comparison to WT cells (Fig. 23d), but did not correlate with the FOXO3 protein levels (Fig. 24a). It is worth noting that after treatment of MPS IIIB cells with resveratrol, the FOXO3 protein levels increased in comparison to untreated MPS IIIB cells.

**Fig. 25:**
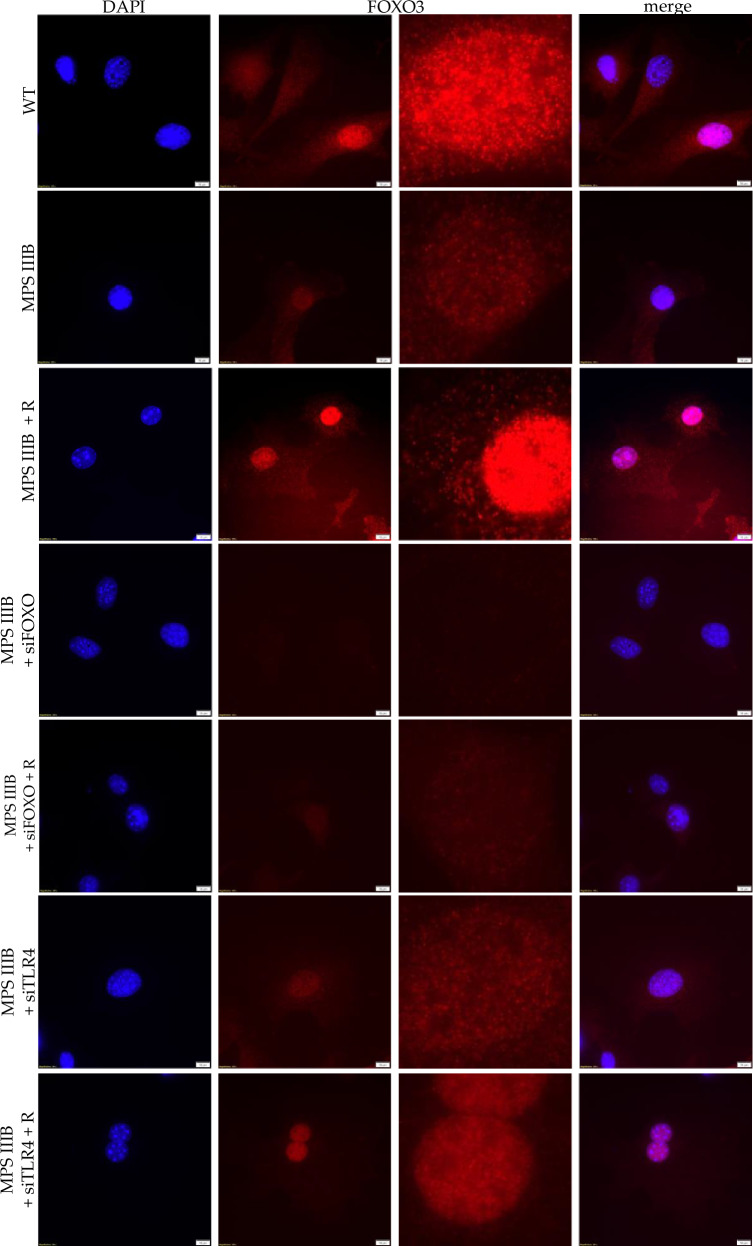
Nuclear translocation of the FOXO3 protein from cytoplasm to nucleus. Images showing WT cells, MPS IIIB cells and MPS IIIB cells treated with resveratrol; MPS IIIB cells treated with siFOXO3 and MPS IIIB cells treated with siFOXO3 and resveratrol; and MPS IIIB cells treated with siTLR4 and MPS IIIB cells treated with siTLR4 and resveratrol. Scale bar, 10 µm at 100× magnification.

As there was no significant difference between WT and MPS IIIB cells, we decided to visualize translocation of the FOXO3 protein to the nucleus, which may explain the disturbance of autophagy in MPS IIIB mice (Figs. 14–16) and cultured fibroblasts (Fig. 23a). As expected, there was a higher colocalization rate of FOXO3 with the nucleus after treatment of cell cultures with resveratrol, confirming that resveratrol activates autophagy though the FOXO3-dependent mechanism (Figs. 24b and 25). There was also decreased efficiency in the translocation of FOXO3 to the nucleus in MPS IIIB cells in comparison to WT cells. According to the proposed correlation between TLR4 and FOXO3, the level of *FOXO3*-derived mRNA increased after treatment with *TLR4*-specific siRNA (Fig. 23d), with no difference in the FOXO3 protein level (Fig. 24a) and no difference in the colocalization of FOXO3 with the nucleus (Figs. 24b and 25). Treatment of cells with resveratrol together with *TLR4*-specific siRNA resulted in an increased efficiency of translocation of FOXO3 into the nucleus in comparison to treatment with *TLR4*-specific siRNA alone in MPS IIIB (Figs. 24b and 25). After simultaneous treatment of cells with *FOXO3*-specific siRNA and resveratrol, there were no statistically significant differences between FOXO3 abundance in the nucleus in comparison to cells treated with the *FOXO3*-specific siRNA alone in MPS IIIB (Figs. 24b and 25).

## Discussion

Resveratrol has been shown to possess various biological properties, such as anti-inflammatory, antioxidant and neuroprotective activities^25^. As many as 175 potential disease-related genes have been identified as potential resveratrol targets^26^. Resveratrol has autophagy-inducing properties, acting through many pathways: (1) by inhibiting mTOR kinase, (2) by inhibiting the expression of the *Bcl2* gene and (3) by activation of sirtuin^27^. Owing to its multiple mechanisms of action, resveratrol was used in experimental therapies for neurodegenerative diseases, carried out in animal and cellular models. In particular, it was investigated in studies on the removal of toxic proteins in disease models characterized by protein aggregation^28^. Research on a cellular model of Huntington’s disease showed that resveratrol, by inducing autophagy, stimulated mutant huntingtin degradation, which resulted in cell protection against dopamine-induced toxicity^29^. In studies on Parkinson’s disease, resveratrol reduced the accumulation of the α-synuclein aggregates, together with a reduction of neuroinflammation and oxidative stress in a mouse model^30^. The neuroprotective effects of resveratrol have also been noted in many models of Alzheimer’s disease, where improvements in behavior have been shown in animals, and an increased proliferation of cells followed stimulation of the SIRT1, AMPK and mTOR signaling pathways, which led to a reduction in the number of amyloid aggregates^31,33^.

Here, we tested a hypothesis on the possible therapeutic effects of resveratrol in Sanfilippo disease. This hypothesis has been proposed recently^27^ on the basis of previous findings and known activities of this compound. In our study, we used the mouse model of MPS IIIB bearing a homozygous knockout mutation in the *Naglu* gene.

We demonstrated that resveratrol supplementation ameliorates the behavioral abnormalities observed in a mouse model of MPS IIIB. Both MPS IIIB males and females exhibited hyperactivity and stereotypy, resulting from increased responses to stress stimuli, in contrast to their WT littermates, while this behavior was corrected after treatment with resveratrol. The therapeutic effect was so efficient that these mice were practically indistinguishable from the control animals at the final stage of the experiment. According to previous observations^61^, *Naglu*-knockout mice at 4–5 months of age do not show any anxiety dysfunction. The only abnormalities in the behavioral pattern they present concerns hyperactivity, which was particularly severe in females. In our experiments, hyperactivity (horizontal and vertical movements), as well as increased stereotypy (ambulatory movements), were evident as early as at 5 weeks of age. In turn, high levels of anxiety, manifested with the shorter time the animals spent in the central squares of the open field, low levels of exploration and longer time in immobility, were also already evident when they reached week 5. These disturbances affected mice of both sexes. The differences in our results compared to those previously reported^61^ may be due to the different experimental design. To avoid the influence of the results of one test on the other, we used a time interval of 24 h between tests. In addition, the tests were conducted in a special order: from the less stressful (analysis in the actometers) to the more stressful (open field test). The time at which the analysis is conducted is also an important issue that can differentiate the results obtained. In the case of our experiments, the locomotor activity analysis took 10 min. This is the optimum time to assess the spontaneous/natural behavior of the rodents. The potential of resveratrol, which has been described extensively in the literature for the improvement of various types of mental disorders, such as depression and anxiety, induced in preclinical studies using different animal models^62^, was also confirmed in our experiments by normalizing hyperactivity and ameliorating the stress response in a mouse model of MPS IIIB.

During the experiments with animals, we observed some sex-related differences in MPS IIIB mice in comparison to WT mice. There were higher total GAG concentrations in male urine than in female MPS IIIB mice during the final week. This did not correlate with behavioral analysis as the hyperactivities of male and female MPS IIIB mice were similar. The only difference was seen in the immobility time; female mice were more stressed, spending more time in the freezing position during the open field experiment. The peripheral immune response was at a similar level in both sexes, but when analyzing several parts of the brain, the changes were more evident. This can be exemplified by the more intensive inflammation in the cortex region of CG2 (higher TNF level) in females; however, in this parameter we also observed a correction after resveratrol treatment in females. The pIRAK1/IRAK1 ratio was consistently upregulated in both male and female MPS IIIB mice in comparison to WT, with the same response to resveratrol; this correlated with the immune response in the peripheral system and CNS. On the other hand, levels of AMPKα were decreased only in MPS IIIB females, with no difference in male mice between MPS IIIB and WT groups.

When analyzing the molecular mechanisms of autophagy in MPS IIIB, sex-dependent differences were evident and should be interpreted in an organ-specific manner, as presented in Supplementary Table 1. Notably, in male mice, there was a general downregulation in the levels of key autophagy-related proteins across tissues. In the brain, spleen and liver, levels of TFEB, SIRT1 and EIF4EBP1, were all decreased compared to WT controls, with increased levels of LC3-II, SQSTM and FOXO3, indicating a widespread impairment of both autophagy initiation and lysosomal function. By contrast, female mice exhibited a more complex pattern. In the brain, the levels of TFEB were increased, potentially reflecting a compensatory activation of autophagy. However, this increase was not uniformly observed across all markers, as levels of LC3-II, SQSTM, EIF4EBP1, RPS6K, SIRT1 and Beclin1 remained unchanged. In the spleen, TFEB and LC3-II levels were elevated with decreased abundance of SIRT1, supporting possible organ-specific activation. In the liver, the majority of investigated markers remained stable, except for a decrease in LC3-II abundance and an increase in SQSTM levels in females, indicating that autophagy disruption in females may be more localized than systemic.

These differences probably contribute to the sex-specific aspects of autophagy regulation observed in MPS IIIB. However, the therapeutic response to resveratrol was remarkably consistent across nearly all examined tissues, with evident induction of autophagy in both sexes. In male MPS IIIB mice, where autophagy markers were broadly suppressed, resveratrol treatment robustly restored levels of these proteins in the brain, spleen and liver, aligning with improvements in behavioral and inflammatory outcomes. In female mice, despite elevated baseline levels of TFEB and LC3-II in certain tissues, such as the brain and spleen, resveratrol still led to further enhancement of autophagy, as assessed by determination of levels of relevant proteins, suggesting that this pathway remained responsive to pharmacological activation.

Together, these results indicated that while male MPS IIIB mice display more pronounced baseline autophagy impairment, both sexes benefit from resveratrol treatment. On the other hand, MPS IIIB females require higher doses of resveratrol to achieve a high efficacy of treatment with this polyphenolic molecule. The consistency of the response across tissues supports resveratrol as a broadly effective autophagy-modulating therapy, though underlying sex-related differences in pathway regulation may still influence the magnitude or dynamics of the therapeutic effect.

Over the past few years, there have been reports suggesting that resveratrol not only has a therapeutic effect but also a protective action when ameliorating various anxiety behaviors or autism spectrum disorders in animal models^63,64^. However, it was suggested that the efficacy of resveratrol may differ significantly between females and males^65^. Nevertheless, our study confirmed the therapeutic efficacy of this compound in improving behavioral disturbances in MPS IIIB mice of both sexes. Behavioral pattern disturbances in the mouse model of MPS IIIB are a consequence of the accumulation of HS, as well as of inflammation that develops not only in the periphery but also in CNS. This disturbs neuronal activity and has a negative impact on the animals’ behavior, which deteriorates, especially as a result of exposure to stress factors, creating a closed loop that intensifies the devastating symptoms observed in the course of this disease.

What are the molecular bases of the differences in both basic regulations of immunological processes and autophagy and the response to resveratrol treatment between MPS IIIB males and females? The most straightforward hypothesis is related to hormonal differences between the two sexes. Hormones are global regulators of metabolic pathways, thus they affect various processes, including immune response and neuroimmune communication, as well as the course of various diseases, including neurological disorders^66–68^. In the case of Sanfilippo disease, reports on the sex-related differences are scarce; however, recent studies indicated that receptors of some sex-related hormones, such as membrane estrogen receptor 1 (GPER1) and oxytocin receptor (OXTR), can directly interact with HS (which accumulates in MPS III) when GAG occurs at relatively high concentrations^69^. Such interactions lead to the formation of aggregates of GAG–GPER1 and GAG–OXTR complexes, thus inactivating these receptors. This can cause disruptions of specific signal transduction processes and severe disturbances in cellular and organismal metabolic processes^69^. Since there are significant differences in the levels of estrogens and oxytocin between females and males, one might easily imagine different effects related to inactivation of receptors of these hormones due to HS-dependent formation of their aggregates. More studies are required to understand the molecular details of these regulations, but if there are more such hormone-related and GAG-dependent dysregulations (which appears to be a likely scenario), one might suggest that they contribute significantly to the observed differences between males and females in the course of MPS IIIB. A similar explanation could be proposed for the differences in the required doses of resveratrol to obtain correction of physiological, immunological and behavioral processes in MPS IIIB male and female mice. While the dosage of 50 mg/kg/day was sufficient to achieve a high efficacy in MPS IIIB males^32^, females required the administration of 150 mg/kg/day of resveratrol to ameliorate the disease symptoms (in this work). In this light, it is important to note that administration of resveratrol to postmenopausal women at the dose of 1 g/day resulted in increased concentrations of sex steroid hormone binding globulin (SHBG), as well as in elevated levels of urinary 2-hydroxyestrone^70^. In fact, the results of both in vitro and in vivo studies suggested that resveratrol might significantly modulate endocrine regulation, especially through the estrogenic effects^71^. Such an activity of resveratrol may considerably influence its efficacy in the treatment of MPS IIIB females relative to that in males.

Despite intensive research, the pathomechanism of MPS is still not fully understood. One of the factors influencing the development of the disease is excessive activation of innate and adaptive immunity and chronic inflammation^72^. The progressive accumulation of HS induces an innate immune system response, which in combination with the secondary aggregation of other molecules, such as gangliosides, impairs lysosome function and enhances the release of proinflammatory cytokines as well as other inflammatory mediators. So far, it has been postulated that this mechanism is mediated by the TLR4 receptor^73^. However, our results suggest that this pathway may not only be different but may also vary in males and females. Two cytokines, IL-1 and TNF, could be crucial in the chronic inflammation in MPS IIIB. These cytokines are released by activated lymphocytes and also by cells of the nervous system, microglia and astrocytes. Their increased secretion was described not only in peripheral blood, but also in the spleen or liver in MPS I, MPS II, MPS IIIA, MPS IIIB and MPS IVA mouse models and patients^74^. By binding to the TNFR1 receptor, TNF mediates activation of proinflammatory pathways, the induction of programmed cell death or the release of prostaglandin E2. Therefore, it is involved in the so-called peripheral symptoms, consisting of increased pain sensation resulting from chronic inflammation, deterioration of physical function and musculoskeletal dysfunction. They are observed not only in animal models, but especially in patients. In turn, IL-1 is thought to be responsible for neuroinflammation and the consequent behavioral abnormalities^73^. However, our study suggests that the role of TNF is not necessarily limited to the induction of peripheral inflammation and the consequent disease symptoms. Elevated levels of this cytokine in key brain areas, determining motor and anxiety responses, showed that its importance in CNS cannot be marginalized. It is also noteworthy that resveratrol therapy not only abolished peripheral inflammation, but also reduced TNF levels in the brains of male and female MPS IIIB mice, improving behavior, an indirect indication linking the role of this cytokine to modulation of the activity of important neuronal circuits. The brains of male and female MPS IIIB mice in the non-resveratrol-supplemented group not only showed neuroinflammation in the cortex and amygdala, but also nonphysiological activation of these brain areas, evidenced by high levels of the c-FOS protein. These results are particularly relevant in the light of increased blood–brain barrier permeability or chronic neuroinflammation, leading to neurodegeneration, which has already been described in 2–3-month-old *Naglu*-knockout mice. The lesions or extensive damage involved axons and dendrites, especially within the cortex and subcortical structures, which include the amygdala^61^.

Interestingly, the results presented in this study, together with the above cited previous studies, corroborate the recently proposed unification of the biology of neurodegeneration in lysosomal storage diseases, a group of severe inherited metabolic diseases of which Sanfilippo disease belongs to. Namely, it was indicated that the secondary storage of various protein aggregates (such as α-synuclein, β-amyloid, hyperphosphorylated tau protein and others) is common in this group of diseases and might significantly contribute to neurodegeneration processes^75^. To explain such a common phenomenon, it was proposed that there is a specific intracellular network leading to CNS neurodegeneration^75^. According to this model, lysosomal dysfunction causes downstream effects, resulting in feedback loops that impair vesicular transport and autophagy. In fact, such impairments have been demonstrated recently for the former^76^ and latter^31^ processes (including the results presented in this study). The primary cause of these dysregulations appear to be disturbances in the control of expression of hundreds of genes in MPS cells^77^, most probably owing to initial impairment of activities of regulatory proteins and/or their genes^78^. The possible influence of primary GAG storage on the disruption of the gene regulation processes through inactivation of specific receptors owing to their interactions with highly abundant GAGs and the formation of inactive aggregates has been confirmed experimentally in the cases of OXTR and GPER1^69^. When disruption of vesicle trafficking and autophagy (as mentioned above) takes place, secondary accumulation of different macromolecular compounds occurs in lysosomes due to inefficient delivery of substrates and their impaired degradation. The latter dysfunction can be further enhanced as substrate accumulation in the lysosome leads to its alkalinization, thus losing the optimal pH for activities of lysosomal enzymes. Moreover, under such conditions, calcium efflux from the lysosome is reduced, which results in mitochondrial function disturbance that causes, in turn, mitophagy initiation. However, owing to impaired autophagy stimulation, this process is ineffective, leading to further accumulation of faulty organelles and proteins, triggering neurodegeneration if the process occurs in neurons^75^.

The concentration of urinary GAGs, monitored over time in this study, indicated the effects of resveratrol on the accumulation of these compounds. The concentration of GAGs in the urine of MPS IIIB and control mice was initially at a similar level, but increased with age in the group of MPS IIIB mice treated with water. Importantly, in the group of MPS IIIB mice supplemented with resveratrol, it remained at a level similar to that measured in control mice, indicating the involvement of resveratrol in the degradation of GAGs. The activity of resveratrol to remove excessive and toxic compounds has already been studied in models of different diseases that are associated with protein aggregation. In one such study^79^, it was shown that in a cellular model of Huntington’s disease (the neuroblastoma-SY5Y line), resveratrol (acting through the induction of autophagy) was involved in the degradation of mutant huntingtin, contributing to the reduction of toxic effects in cells. Furthermore, the use of resveratrol in combination with β-cyclodextrin in studies with a cellular model of Parkinson’s disease resulted in stimulation of the degradation of α-synuclein aggregates^80^. The neuroprotective effect of resveratrol, linked to the degradation of β-amyloid, the accumulation of which is the major cause of Alzheimer’s disease, was also described^81^.

An intriguing observation in our experiments was that behavioral changes could be observed in MPS IIIB mice before increased urinary GAG levels could be detected. Moreover, the effects of resveratrol could also be determined irrespective of a lack of obvious urinary GAG storage. Somewhat similar effects were found for cytokines. Therefore, it is tempting to hypothesize that Sanfilippo syndrome symptoms are not necessary the sole effects of massive GAG accumulation. Interestingly, lysosomes with aggregates and deposits were observed in the brains of MPS IIIB mice at the age of 6 weeks, which is several weeks before elevated GAG excretion to the urinary tracts can be detected^82^. Moreover, at the same age of these mice, microglia activation, astrocytosis and intense inflammatory processes, as well as histopathological changes, were reported^82^. Furthermore recently published results indicated that behavioral changes in another Sanfilippo diseases mouse model, MPS IIIA, may be due to increased proliferation of mesencephalic dopamine neurons, and not necessary to dysfunctions of lysosomes^83^. Indeed, it was proposed that disturbed functions of HS might be responsible for pathological changes in dopamine activity, while elevated GAG levels do not play a crucial role in this pathomechanism^83^. In this light, one can propose that our results are fully compatible with those previously published, which indicated that GAG storage is not necessary the only pathomechanism of MPS^75^. Such a hypothesis is corroborated by recent reports showing that the expression of hundreds of genes and many cellular processes is significantly disturbed in MPS cells, which is not necessary in simple correlation with the levels of GAG storage^77,84–86^.

Relatively high dosages of resveratrol (150 mg/kg/day) were used in this study. If resveratrol were the approved drug, this would mean a dose of several grams per days per patient, which is a high amount. However, although significantly lower doses have been used in clinical trials performed so far^87^, this dose was chosen because it was demonstrates that, despite the ability of resveratrol to cross the blood–brain barrier^34^, the efficiency of this crossing is low, significantly lower than that of many other polyphenolic compounds^88^. Therefore, it remains to be determined what is the lowest effective dose of resveratrol in improving symptoms of Sanfilippo disease. One might also consider that increasing the efficiency of delivery of resveratrol to the brain may be of special interest. Indeed, some recently published reports provide hope that various approaches can be successful in this matter^89,90^.

The results of this study corroborate the previous proposal that resveratrol can stimulate the autophagy process^32^. Here, we sought to determine the molecular pathway of resveratrol-mediated autophagy induction in MPS IIIB. Evidently, under these conditions, resveratrol stimulated autophagy by at least two pathways, mTOR dependent and requiring FOXO3. These conclusions were made based on the analyses of the levels of proteins involved in the specific pathway, as well as the results of experiments silencing the expression of the *FOXO3* gene. Undoubtedly, resveratrol stimulated translocation of FOXO3 from the cytoplasm to nucleus due to dephosphorylation of this protein.

It was previously reported that TLR4 activation can inhibit autophagy through FOXO3^91^. Thus, we hypothesized that excessive TLR4 activation by HS may contribute to impaired autophagy in MPS IIIB. Silencing the gene encoding TLR4 reduced autophagy efficiency, suggesting an interplay between autophagy and the immune response. However, silencing *TLR4* gene expression did not prevent resveratrol-enhanced translocation of FOXO3 into the nucleus. Therefore, the interconnection between the immune system and autophagy in MPS IIIB appears to be highly complex and requires further investigation.

## Supplementary information

Supplementary Information

## Data Availability

Raw data and all materials are available from the authors upon request.
