# Author Correction: TFEB, FOXO3 and TLR4 in resveratrol-induced autophagy in a mucopolysaccharidosis IIIB mouse model

**DOI:** 10.1038/s12276-026-01694-3

**Published:** 2026-03-17

**Authors:** Estera Rintz, Magdalena Podlacha, Lidia Gaffke, Grażyna Jerzemowska, Zuzanna Cyske, Karolina Pierzynowska, Grzegorz Węgrzyn

**Affiliations:** 1 Department of Molecular Biology, Faculty of Biology, University of Gdańsk, Gdańsk, Poland; 2 Department of Animal and Human Physiology, Faculty of Biology, University of Gdańsk, Gdańsk, Poland

**Keywords:** Molecular neuroscience, Macroautophagy, Mechanisms of disease

Correction to: *Experimental & Molecular Medicine* 10.1038/s12276-026-01643-0, published online 05 February 2026

After online publication of this article, the authors noticed errors in Figs. 5 and 8.

Figure 5: An incorrect figure was included in the final version of the manuscript, and this was not detected during the final checks. At present, Figs. 4 and 5 are identical in the published online version. Correct Fig. 5 should have appeared as below.

Incorrect figure 5
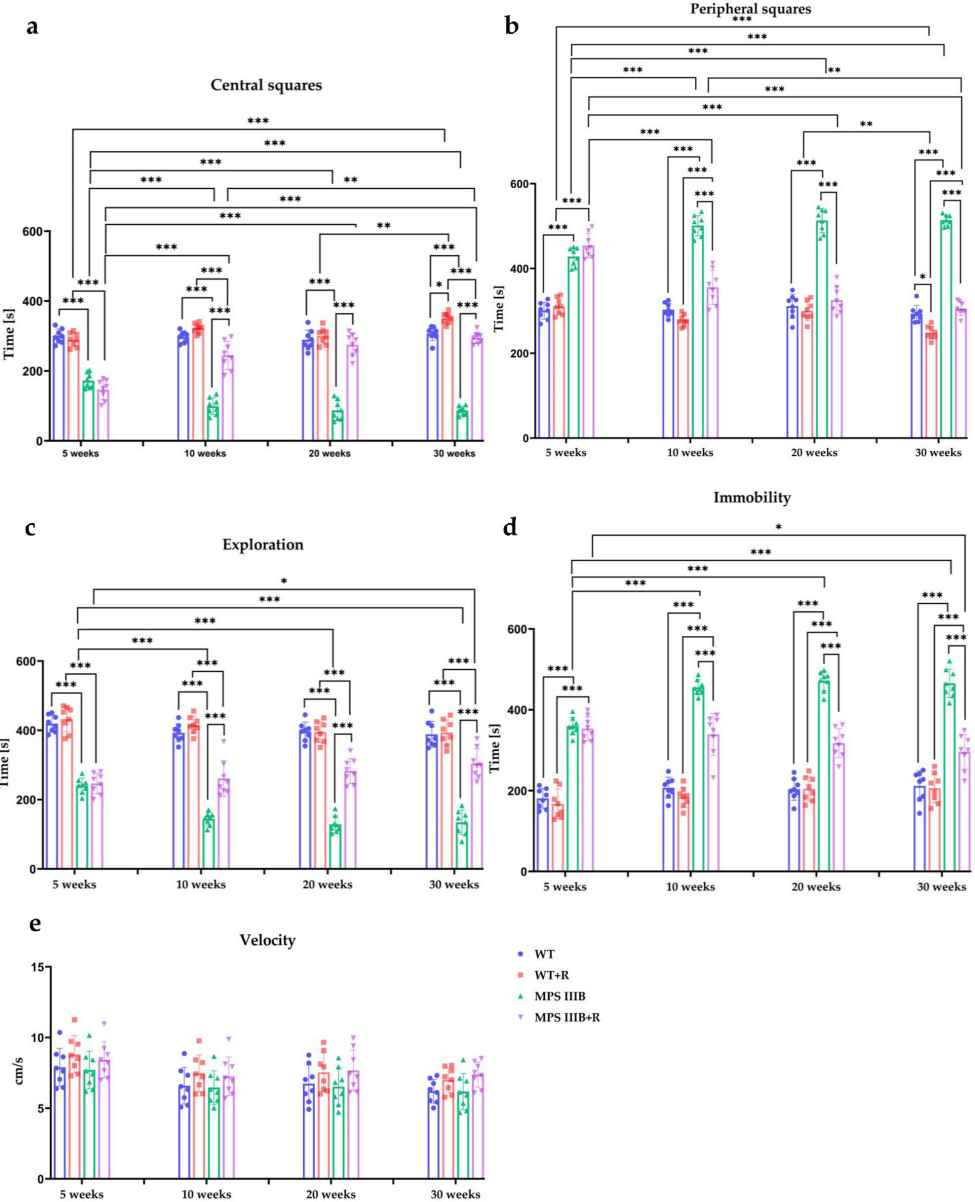


Correct figure 5
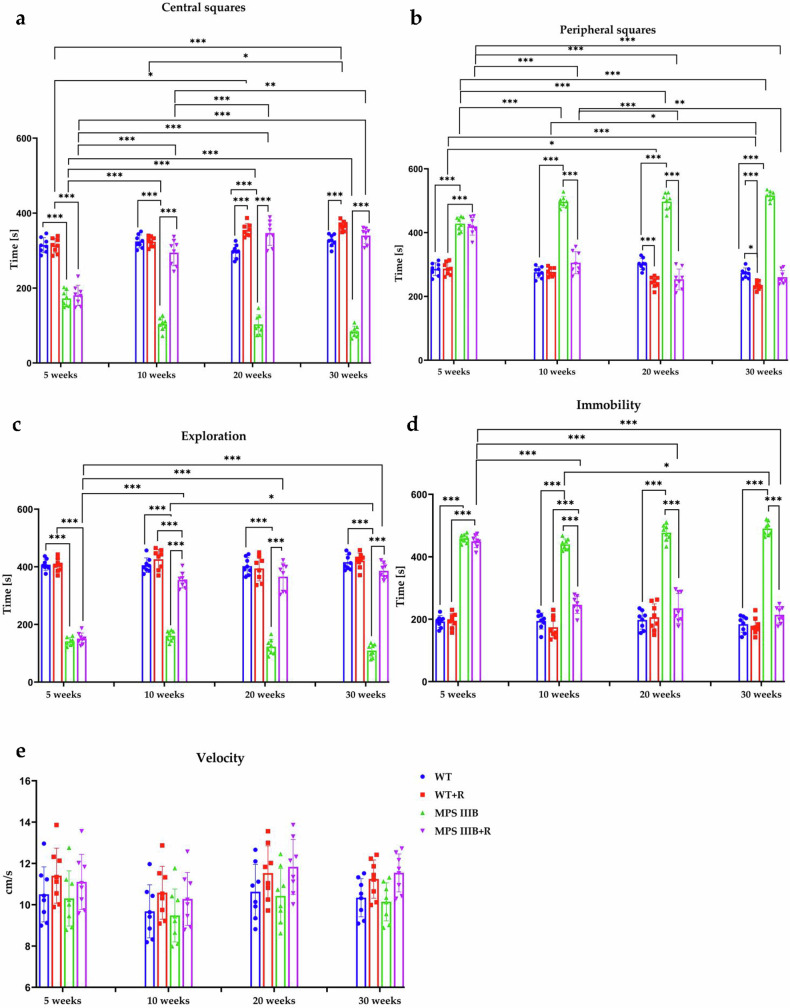


Figure 8: At present, the representative picture of MPS IIIB males in section “a” and the picture of MPS IIIB males in section “b” are the same at the moment and were not detected during the final checks. The correct Fig. 8 should have appeared as shown below.

Incorrect figure 8
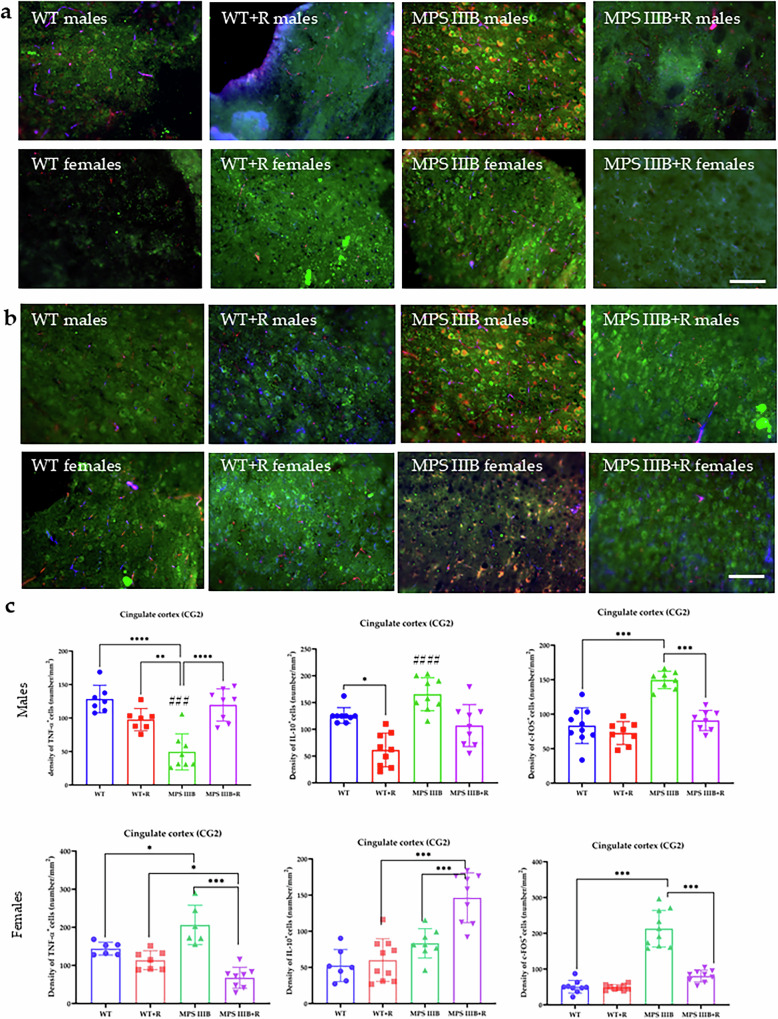


Correct figure 8
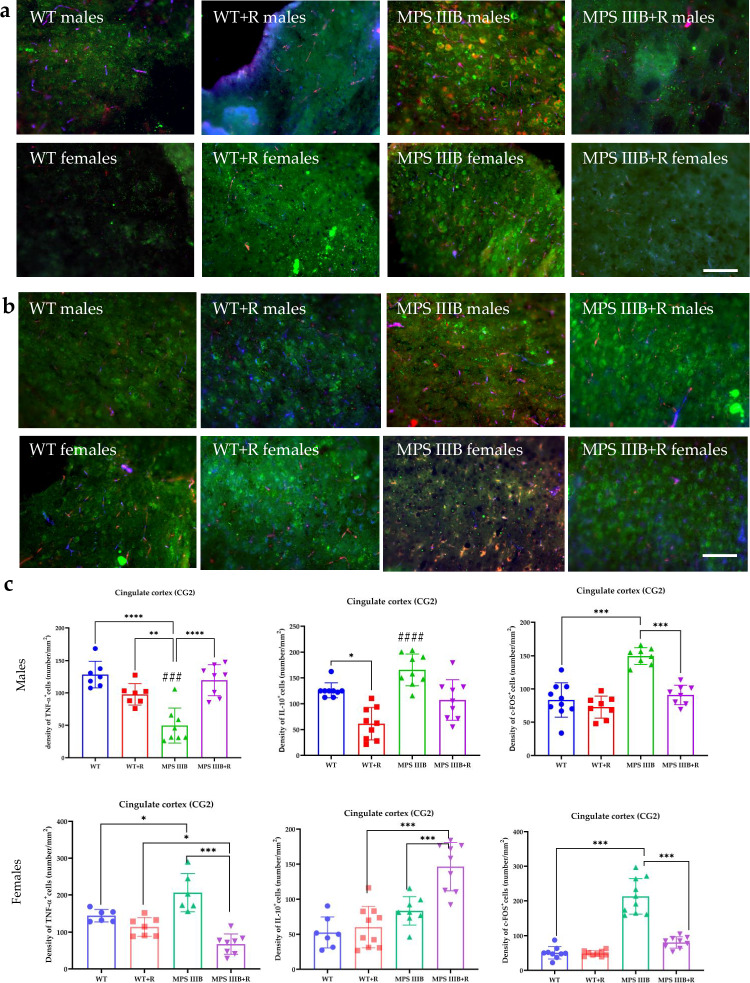


The authors apologize for any inconvenience caused.

The original article has been corrected.

