# Supplementary material for: TFEB, FOXO3 and TLR4 in resveratrol-induced autophagy in a mucopolysaccharidosis IIIB mouse model
Source: Exp Mol Med. 2026 Feb 5;58(2):436–71. doi: 10.1038/s12276-026-01643-0 (PMC12992813; doi:10.1038/s12276-026-01643-0)
Supplement: Supplementary file 1 — Supplementary Information [file 12276_2026_1643_MOESM1_ESM.pdf]

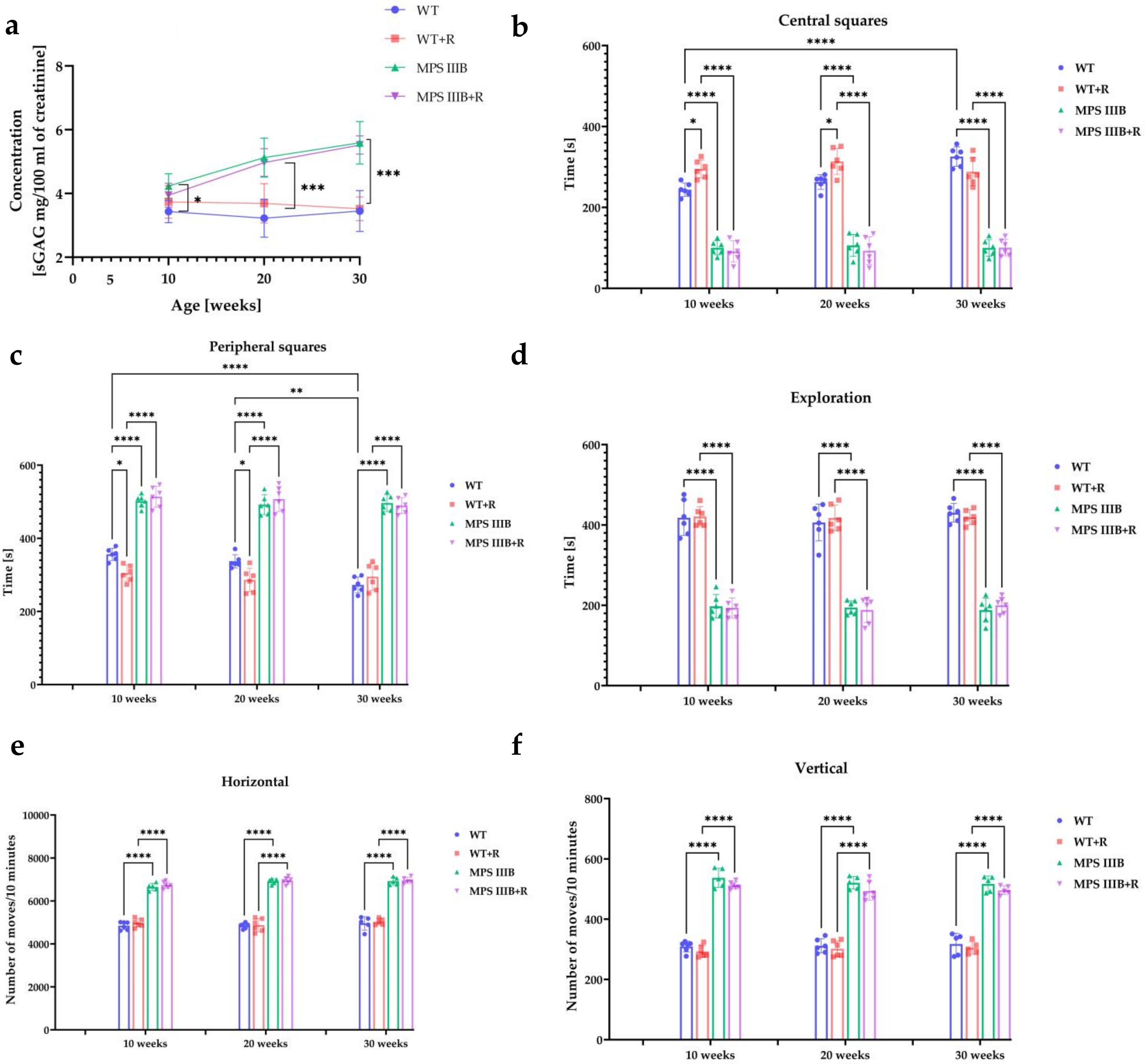

Supplementary Fig. 1. Efficacy of resveratrol treatment with 50mg/kg in females mice. (a) Urinary GAG levels; (b-d) Anxiety-related behavior measured in open field test. (e-f) Locomotor activity measurment. Results are presented as mean ± standard deviation (SD) (n=5-6). Statistically significant differences are indicated by asterisks: *$p < 0.05$; **$p < 0.01$; ***$p < 0.001$; ****$p < 0.0001$.

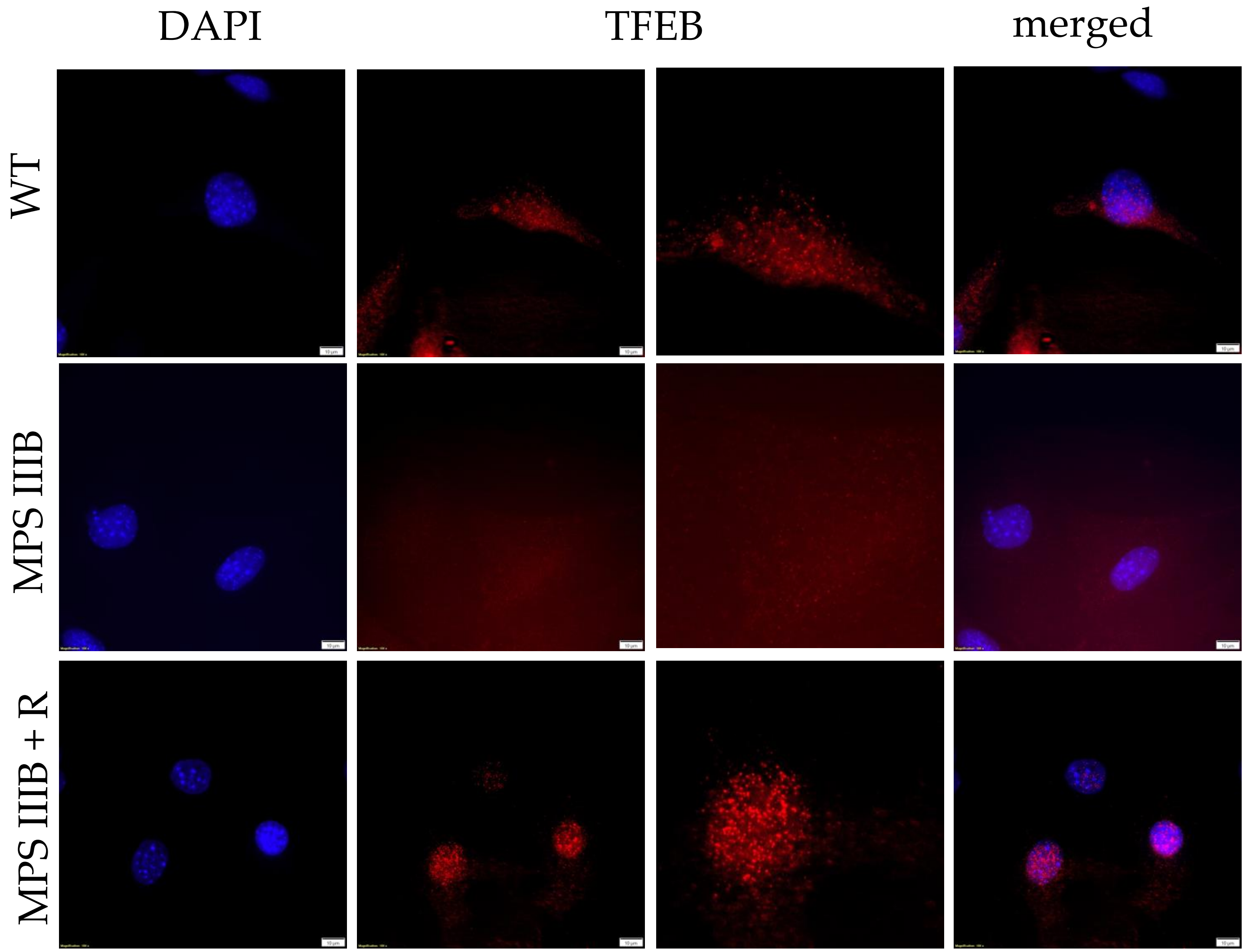

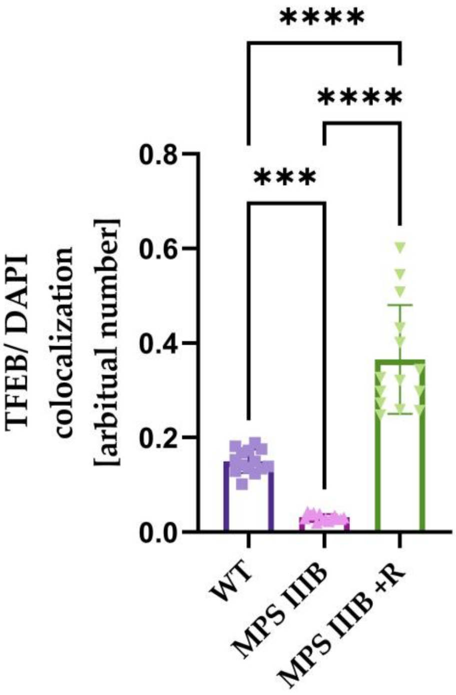

Supplementary Fig. 2. Nuclear translocation of the TFEB protein from cytoplasm to nucleus. Panels of: WT cells; MPS IIIB cells; MPS IIIB cells treated with resveratrol. 100x magnification with 10 µm scale bar. Statistical analysis of Pearson's correlation coefficient of nucleus fluorescence. Results are shown as mean values ± SD (n=3). Statistically significant differences are indicated by asterisks: ***$p < 0.001$; ****$p < 0.0001$.

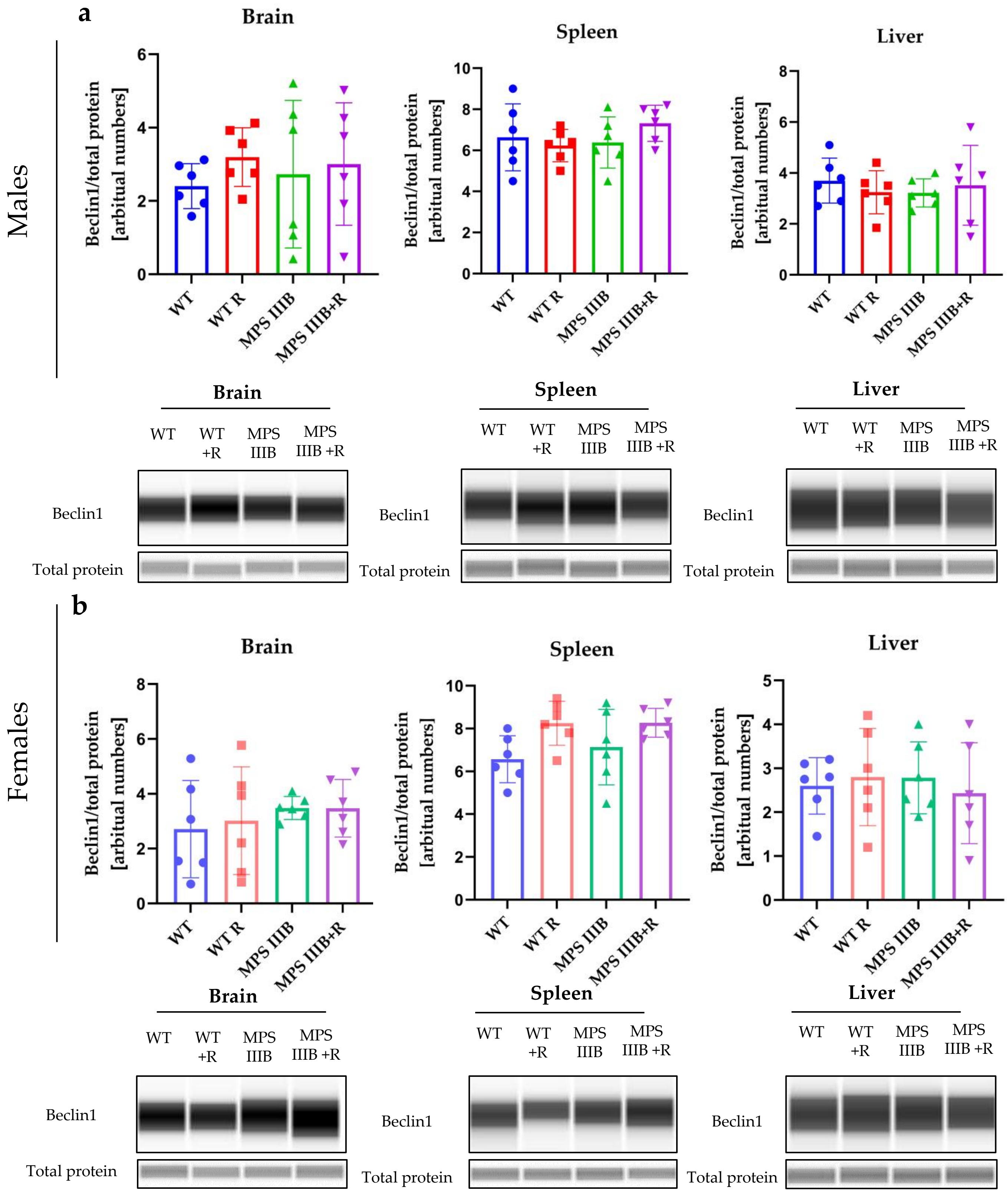

Supplementary Fig. 3. (a) Representative western blots of Beclin-1 protein level with statistical analysis in brain, spleen, and liver of male mice group; (b) Representative western blots of Beclin-1 protein level with statistical analysis in brain, spleen, and liver of female mice group. Results are presented as mean ± standard deviation (SD) (n=5-6). There was no statical significance.

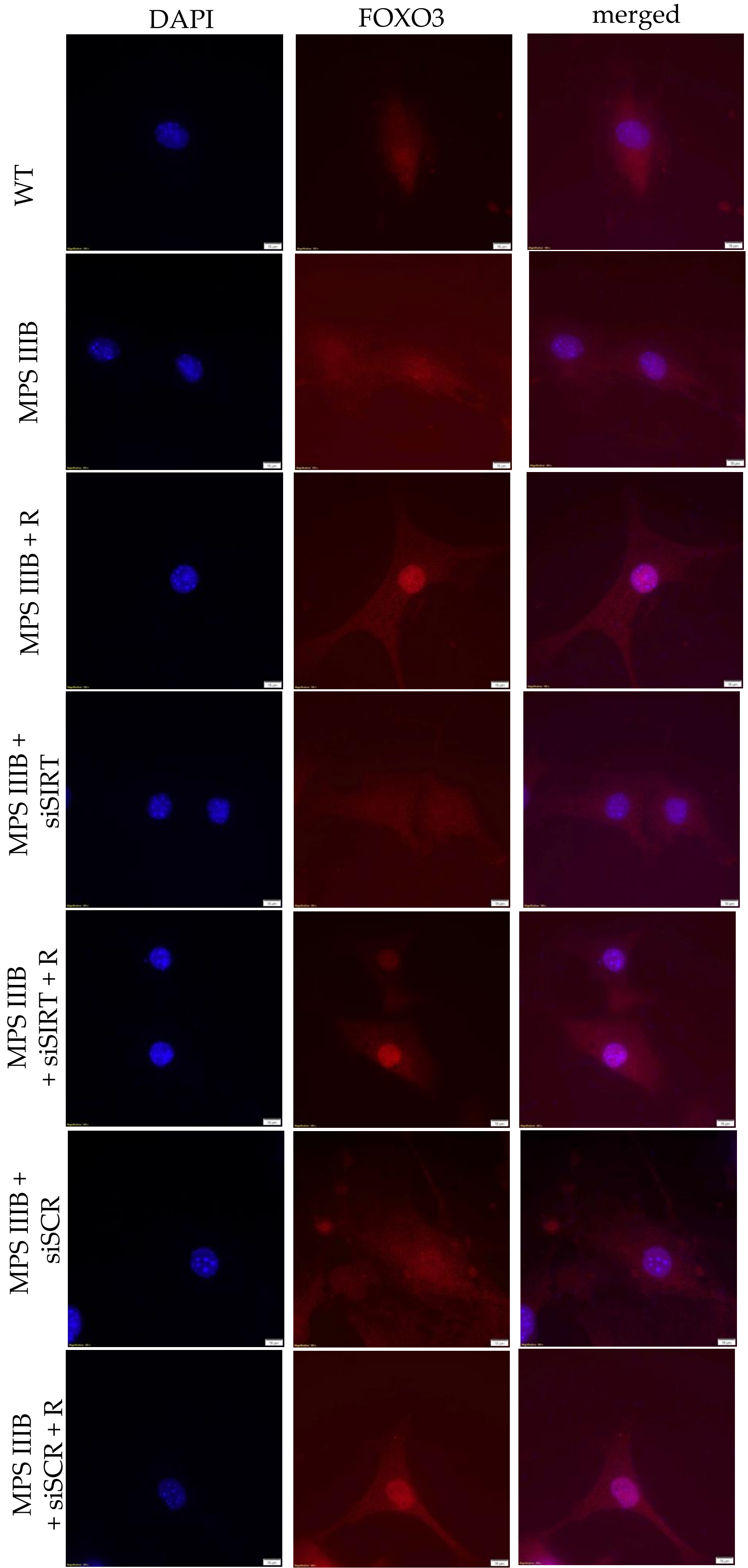

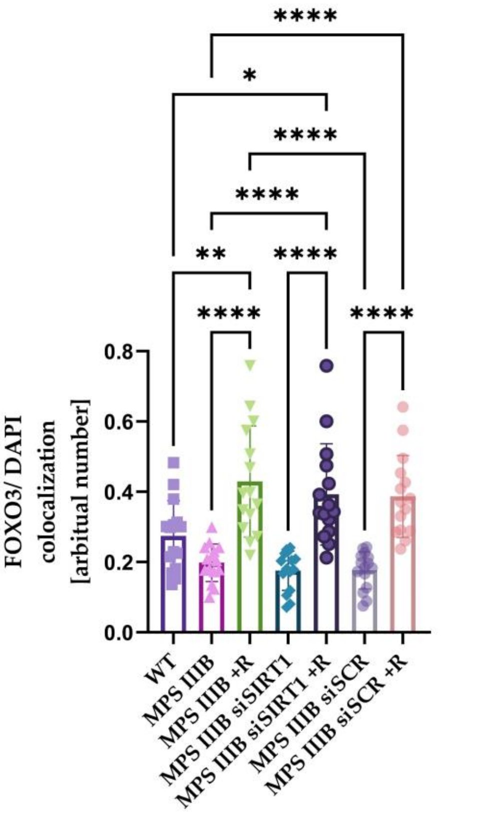

Supplementary Fig. 4. Nuclear translocation of the FOXO3 protein from cytoplasm to nucleus. Panels of: WT cells; MPS IIIB cells; MPS IIIB cells treated with resveratrol; MPS IIIB cells treated with siSIRT1; MPS IIIB cells treated with siSIRT1 and resveratrol; MPS IIIB cells treated with siSCR; MPS IIIB cells treated with siSCR and resveratrol. 100x magnification with 10 μm scale bar. Statistically significant differences are indicated by asterisks: *$p < 0.05$; **$p < 0.01$; ****$p < 0.0001$.

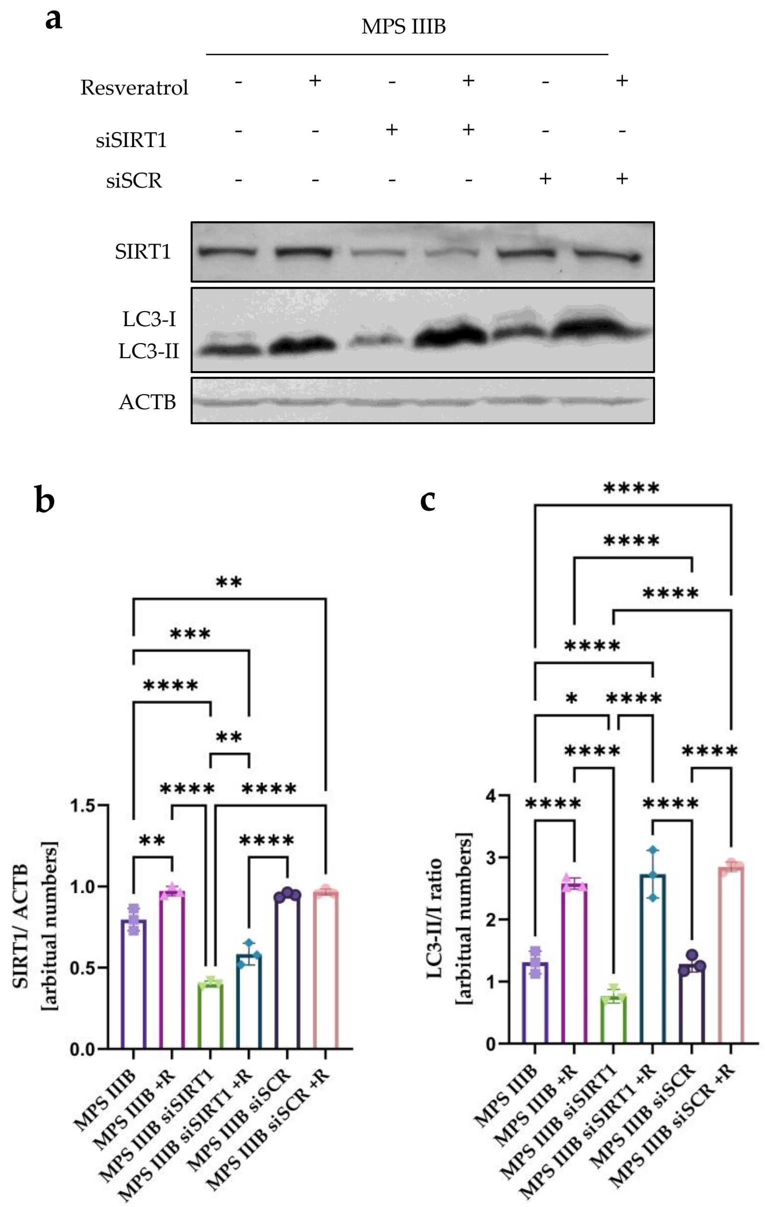

Supplementary Fig. 5. (a) Representative western blots of SIRT1 and LC3-II protein level with statistical analysis in the mouse cells after silencing with siSIRT1 and siSCR (scrambled siRNA); (b) Representative western blots of SIRT1 and LC3-II protein level with statistical analysis in the mouse cells after silencing with siSIRT1 and siSCR; **(c)** Relative SIRT1 mRNA expression, siSCR- scrambled control. Results are presented as mean ± standard deviation (SD) (n=3). Statistically significant differences are indicated by asterisks: *$p < 0.05$; **$p < 0.01$; ***$p < 0.001$; ****$p < 0.0001$.

a

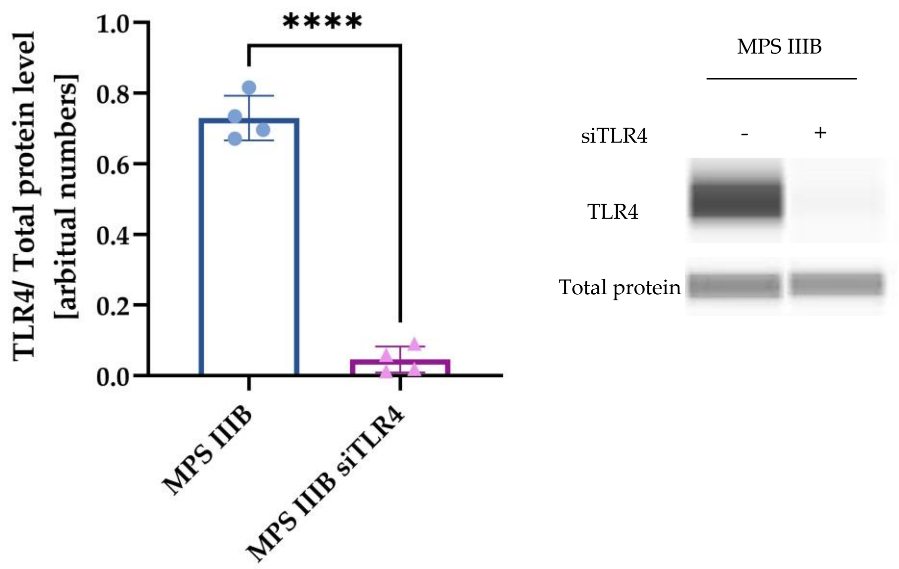

b

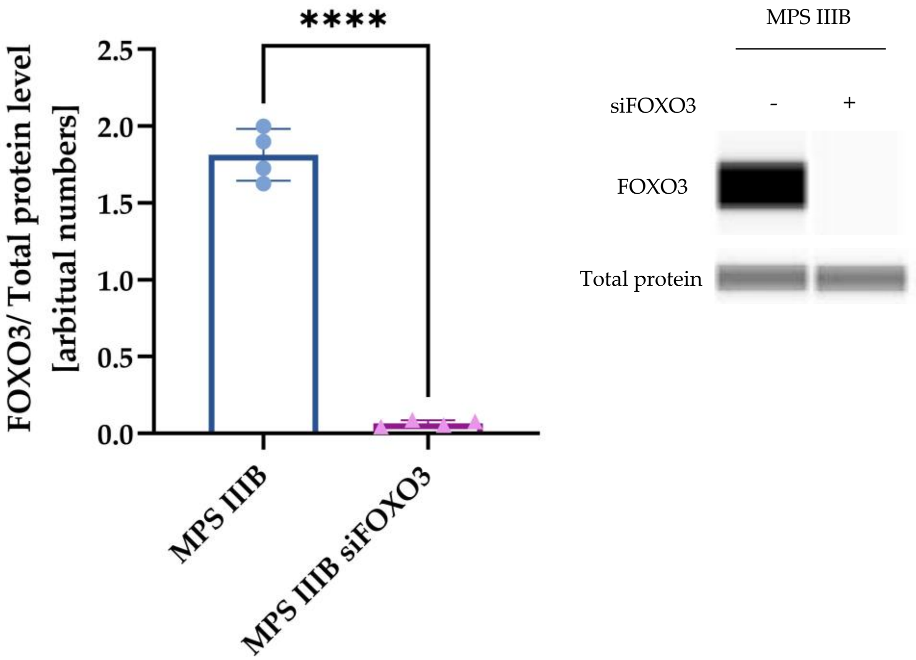

Supplementary Fig. 6. (a) Representative western blots of TLR4 protein level with statistical analysis in the mouse cells after silencing with siTLR4; (b) Representative western blots of FOXO3 protein level with statistical analysis in the mouse cells after silencing with siFOXO3. Results are presented as mean ± standard deviation (SD) (n=4). Statistically significant differences are indicated by asterisks: ****$p < 0.0001$.

Protein	WT vs MPS IIIB						MPS IIIB vs MPS IIIB+R					
	male			female			male			female		
	B	S	L	B	S	L	B	S	L	B	S	L
IRAK1/ pIRAK1	↑			↑			↓			↓		
AMPKα	—			↓			—			↑		
LC3-II	↑	—	↑	—	↑	↓	↑	↑	ns↑	↑	↑	↑
SQSTM	↑	↓	↓	—	—	↑	↓	↓	↓	↓	↓	↓
TFEB/ pTFEB	↓	↓	↓	↑	↑	—	↓	↑	—	↓	↓	↓
EIF4EBP1/ pEIF4EBP1	↓	—	—	—	—	—	—	—	↓	—	↓	↓
RPS6K/ pRPS6K	—	—	—	—	—	—	↓	↓	↓	↓	↓	↓
SIRT1/ pSIRT1	ns↓	—	ns↓	—	↓	ns↓	—	↑	↑	ns↑	↑	↑
FOXO3/ pFOXO3	↑	↑	↑	ns↓	—	↑	↓	↓	↓	↓	—	↓
Beclin1	—	—	—	—	—	—	—	—	—	—	—	—

Supplementary Table 1. Comparison of molecular mechanism of autophagy changes in male and female mice. Abbeveratrion: B-brain; S-spleen; L-liver; ns-not significant; ↑- higher protein level; ↓- lower protein level; — no change.
